# Recent Progress in Multifunctional Graphene-Based Nanocomposites for Photocatalysis and Electrocatalysis Application

**DOI:** 10.3390/nano13132028

**Published:** 2023-07-07

**Authors:** Zanhe Yang, Siqi Zhou, Xiangyu Feng, Nannan Wang, Oluwafunmilola Ola, Yanqiu Zhu

**Affiliations:** 1 State Key Laboratory of Featured Metal Materials and Life-Cycle Safety for Composite Structures, School of Resources, Environment and Materials, Guangxi University, Nanning 530004, China; 2039200206@st.gxu.edu.cn (Z.Y.); 2039200234@st.gxu.edu.cn (S.Z.); 2139200335@st.gxu.edu.cn (X.F.); y.zhu@exeter.ac.uk (Y.Z.); 2 Advanced Materials Group, Faculty of Engineering, The University of Nottingham, Nottingham NG7 2RD, UK

**Keywords:** graphene composites, photocatalysis, electrocatalysis, pollutant degradation, CO_2_ fixation

## Abstract

The global energy shortage and environmental degradation are two major issues of concern in today’s society. The production of renewable energy and the treatment of pollutants are currently the mainstream research directions in the field of photocatalysis. In addition, over the last decade or so, graphene (GR) has been widely used in photocatalysis due to its unique physical and chemical properties, such as its large light-absorption range, high adsorption capacity, large specific surface area, and excellent electronic conductivity. Here, we first introduce the unique properties of graphene, such as its high specific surface area, chemical stability, etc. Then, the basic principles of photocatalytic hydrolysis, pollutant degradation, and the photocatalytic reduction of CO_2_ are summarized. We then give an overview of the optimization strategies for graphene-based photocatalysis and the latest advances in its application. Finally, we present challenges and perspectives for graphene-based applications in this field in light of recent developments.

## 1. Introduction

With the acceleration of urbanization, mankind in the 21st century is facing the two problems of global warming and energy shortage. Among the abundant gases in the atmosphere, CO_2_ is the most important factor determining the trend of the greenhouse effect. The excessive development of fossil energy causes energy shortage and also promotes the excessive emission of CO_2_. Various environmental pollution problems around the world are becoming more and more serious [1]. There are many studies on the reduction of CO_2_, but only a few semiconductors meet the reduction requirements for CO_2_ under sunlight irradiation [2,3,4,5]. The reason is that effective charge separation is crucial for the photocatalytic reduction of CO_2_. A large number of studies have found that the composite material formed by using graphene-doped semiconductors in photocatalysis significantly improves the effect of the photocatalytic reaction and greatly improves the reduction efficiency [6,7,8,9,10]. In particular, graphene can generate specific fuels in the photocatalytic reduction of CO_2_ through its unique electron collection and transfer capabilities [11,12].

Water resources are important survival resources. According to statistics, human beings can drink only 3% of the global water resources. With the increase in the world’s population, there is a shortage of water resources in some parts of the world. More seriously, the water environment has also deteriorated, making the water resources available for human use significantly reduced [13]. The deterioration of water resources usually comes from domestic sewage, industrial wastewater, mining wastewater, etc. This sewage contains organic refractory substances, heavy metal ions, etc., which will cause damage to the human body upon drinking and also cause certain damage to the ecological environment [14]. In view of the shortage of water resources and serious pollution [15,16,17,18,19], low-cost and efficient sewage purification methods are of great significance to human beings [20,21,22,23]. Sewage purification can be performed via physical or chemical methods, including filtration, adsorption, boiling, distillation, chlorination, electromagnetic radiation, etc.; however, these methods still contain obvious drawbacks [13,24]. For example, the chlorination of purified water will produce many carcinogens during the process, and the hypochlorite of purified water has strong corrosivity, which further increases its treatment cost [25,26,27].

In recent decades, the use of renewable solar energy technology to remove pollutants has gradually become a research trend [28,29,30]. This is because photocatalysis can produce reactive oxygen species and sterilize and destroy various organic and inorganic pollutants; furthermore, it is cheap, which also shows that photocatalysis is an important research direction for water purification [31]. However, considering the cost and photocatalytic effect, doping graphene is a good method [32,33,34]. Graphene is a single-layer two-dimensional structure formed by the arrangement of carbon atoms. It has a high surface area, optical transmittance, and chemical stability [35]. It is not only an ideal choice for pollutant treatment applications, but because of its biocompatibility and efficient use of photocatalytic efficiency, it has also become a leading candidate in the field of photocatalysis [35]. The structure of graphene is formed by arranging carbon atoms one by one. The internal carbon atoms are bonded by “sp^2^” hybrid orbitals. The normal “pz” orbital of the layer plane of the cabinet carbon atom can form a large π bond throughout the whole layer. There is a strong “s” bond between the two carbon atoms, which greatly enhances its structural strength and flexibility. Based on this hybridization, the carbon atoms connected by graphene “sp^2^” hybridization can be tightly stacked into a single-layer two-dimensional honeycomb lattice structure, with a high specific surface area and good electronic properties [36]. At the same time, the unique band structure also makes graphene have good conductivity and electron mobility [37]. Graphene has a variety of application characteristics. In the field of multifunctional components, it can be used in the tunable terahertz filter/antenna-sensor of graphene metamaterials. The center frequency of the filter and antenna sensor can be adjusted by changing the chemical formula of graphene, and the thickness of the graphene-layered material can be increased to increase the depth of the enhanced resonance effect [38,39].

On the other hand, in the application of three-dimensional absorbers, three-dimensional metamaterial curved ultra-wideband absorbers can exhibit better microwave absorption properties similar to those of reduced graphene oxide (rGO)/MWNTs hybrids [40,41,42,43]. Borah et al. prepared the expanded graphite non-metallic flexible metamaterial absorber by using the linear low-density polyethylene as the substrate material to composite the expanded graphite (EG). Compared with copper-based absorbers, the resonance frequencies of expanded graphite-based absorbers are similar (EG = 11.56 GHz, Cu = 11.73 GHz), but the reflection loss of expanded graphite-based absorbers is much lower than that of copper-based absorbers (EG = −24.51 dB, Cu = −7.32 dB) [44]. The EG material also has the characteristics of heat resistance, low thermal expansion, and oxidation resistance, and it has certain application potential in the field of electromagnetic wave absorbers [45,46]. Singhal studied an ultra-wideband infrared absorption device with an absorption rate of more than 90% and an operating bandwidth of more than 74 THz from 6 THz. This device uses a dielectric material such as graphite as a substrate composite of SiO_2_. Due to the temperature stability of graphene [47], such devices can be well applied in the terahertz and infrared spectral bands in the future [48]. Norouzi et al. proposed a low-cost, simple, and efficient 3D metamaterial ultra-wideband absorber that is insensitive to the incident angle of 60° in the TE mode (Transverse Electric, no electric field in the direction of propagation) and 90° in the TM mode (Transverse Magnetic, no magnetic field in the direction of propagation), and that is not affected by the polarization of the incident wave. This shows that the device is not sensitive to other electromagnetic wave segments under the initial receiving condition setting, and that it has strong anti-interference or shielding ability [49]. The flexible structural characteristics and broadband-absorption capacity, low thermal-expansion coefficient and antioxidant functional characteristics of graphene-based materials confer them great development potential and application prospects in the fields of medical treatment, imaging, and microwave absorption in the future [50,51,52,53,54,55].

In addition to the important application of graphene 3D metamaterials in the field of microwave absorption, some graphene aerogel materials also have outstanding performance in electromagnetic wave absorption [56,57,58,59]. Wu et al. dispersed graphene oxide (GO) uniformly in the chain formed by a polypyridine gel and reduced GO to rGO by the hydrothermal method. After the purification and drying process, using a large amount of distilled water and ethanol, a sponge-like polypyrrole (S-PPy)/rGO aerogel was obtained. This material not only has the lightweight properties of aerogel materials but also has low reflection loss (−54.4 dB at 12.76 GHz) [60]. The graphene@SiC aerogel composites studied by Jiang et al. also have the characteristics of low reflection loss (−47.3 dB at 10.52 GHz) [61]. The special structure of the aerogel material endows it with compressible characteristics, and it shows obvious electromagnetic sensitivity and strong adsorption performance under certain conditions [62,63,64,65]. Wang et al. prepared macroscopic 3D-independent porous all-graphene aerogels with ultra-light density and high compressibility by an in situ self-assembly and thermal annealing process. The ice crystal growth, GO reduction and the restoration of π-conjugation during the freeze-drying process will give the material a 3D structure, thereby obtaining good recoverable compressibility and a strain level of up to 75%, which gives the aerogel a highly sensitive strain response characteristic in volume conductivity. At the same time, the high-temperature-stable graphene composition and large porosity aerogel structure can quickly remove heat during the combustion process, reflecting a certain flame retardancy [66]. Li et al. made full use of the adsorption of aerogels and the hydrophobicity of graphene sheets to synthesize hydrophobic aerogels with high porosity, which can absorb different organic liquids or be used to separate and absorb organic pollutants from water [67]. Hong et al. also studied the selective adsorption of aerogels. On the basis of non-functionalized graphene aerogels with high porosity and hydrophobicity after surface modification, they introduced fluorinated functional groups into the surface of three-dimensional macroporous graphene aerogels by a one-step immersion method to obtain functionalized rGO (F-rGO). This material has the physical properties of low density (bulk density of 14.4 mg cm^−3^), high porosity (>87%), mechanical stability (supports at least 2600 times its own weight), and hydrophobicity (contact angle of 144°). At the same time, the team tested the absorption capacity of F-rGO aerogels for various oils and organic solvents such as pump oil, chlorobenzene, tetrahydrofuran, and acetone. The results show that F-rGO aerogel has excellent adsorption efficiency for various oils and organic solvents, and the adsorption capacity is 34~112 times its weight. The absorption capacity depends on the density of organic solvents. Pre-introduction of fluorinated functional groups can be used to selectively remove oil or take away more O atoms [68]. There are also many examples of supercapacitor applications [69,70,71]. A graphene-based nitrogen self-doped hierarchical porous carbon aerogel was synthesized by Hao et al. using chitosan as the raw material through a carefully controlled aerogel formation–carbonization–activation process. The specific capacitance calculated from charge–discharge measurements using an all-solid-state symmetric supercapacitor was about 197 F g^−1^ at 0.2 A g^−1^ with an excellent capacitance retention of ~92.1% over 10,000 cycles. The energy density reached as high as 27.4 W h kg^−1^ at a power density of 0.4 kW kg^−1^ and 15 W h kg^−1^ at a power density of 20 kW kg^−1^ [72]. In terms of photocatalysis, of the many metal-based materials, an organic-semiconductor has certain applications [73,74], with a long service life and high water decomposition efficiency, but its effect and economy can still be improved. The doping of graphene and graphene-based materials provide a good basis for forming heterojunctions. The doping of graphene brings its own high specific surface area, high carrier migration speed, high conductivity and other characteristics to the doped composite materials, which can effectively solve the problems of material environmental protection and the rapid recombination of photogenerated electron holes and significantly improve the overall efficiency and resource utilization of photocatalysis [75,76]. With regard to the selection of dopants, these are generally divided into metal and nonmetal. Metal can act as an ‘electronic warehouse’ for releasing electrons, fix on some sites of graphene to improve the photocatalytic effect [77,78], or fix on graphene to provide appropriate adsorption and activation to improve the activity of the whole material [79]. Metal doping is quite a good idea, but because the price of metal materials is slightly expensive and some metals are not friendly to the environment, the development of metal-anchored graphene composites is still challenging. Non-metallic doping, single doping or multi-doping have significantly improved the trend of photocatalysis, and can also achieve the same effect as metal doping [80]. N and S doping is quite typical of non-metallic doping; when these non-metallic material co-catalysts are anchored in the active site of graphene, they can limit some metal-atom couplings. In terms of dimensional stacking, the extension of linear one-dimensional conjugated polymers to two- or three-dimensional polymers can significantly enhance exciton dissociation, effectively producing free electrons and abundant reaction sites.

This paper reviews the mechanism of the photocatalytic production of various products and also shows the optimization and promotion of graphene in photocatalysis, as well as the latest research progress of graphene in this field. In the following chapters, we will focus on the effect and mechanism of graphene doping and its different dimensional applications on the improvement of the photocatalytic effect, including photocatalytic water splitting to produce hydrogen, the reduction of carbon dioxide, the degradation of pollutants, etc. Finally, we try to provide an understanding of the current progress, future trends, and challenges of graphene photocatalysis.

## 2. Photocatalytic Water Splitting for Hydrogen Production and Electrocatalysis

In the context of energy shortage and environmental degradation, hydrogen, as a clean energy that can be stored in large quantities, is considered to be the main carrier of future energy and has attracted more and more attention in various fields [81,82,83,84,85,86]. In 1972, the University of Tokyo in Japan used the n-type TiO_2_ semiconductor as an anode and Pt as a cathode to produce a solar photoelectrochemical cell, which realized the photodecomposition of water to produce hydrogen [87]. According to the characteristics of semiconductor light-excitation electron transition and photocatalytic reaction, researchers have developed a device for hydrogen production by the photocomplexation catalytic decomposition of water. Through a series of coupling processes, that is, artificially simulating the process of water decomposition by photosynthesis, the efficiency of hydrogen production is generally 6% [88,89,90,91]. In order to use hydrogen energy to solve the problem of energy shortage or to provide better commercial benefits, technical breakthroughs and more effective materials are needed. At present, graphene-based materials are the better choice, because graphene can be used as a transfer carrier of electrons, reducing the requirement for electrons to pass through the valence band and significantly enhancing the photocatalytic reaction effect [92]. In the next chapter, the mechanism of the photocatalytic reaction and the beneficial optimization strategy of graphene-based photocatalysis will be introduced, mainly from the dimension structure of doping and graphene.

### 2.1. Mechanism

**Figure 1:**
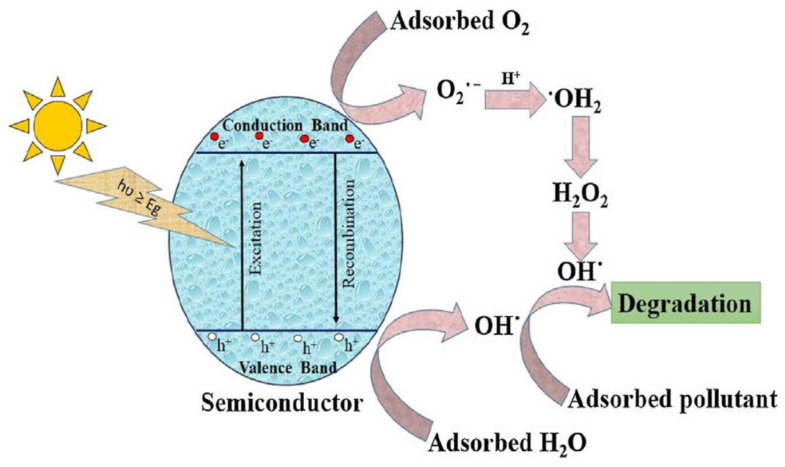
Semiconductor−based photocatalysis perspective. Reprinted with permission from Ref. [93]. Copyright©2018 Production and hosting by Elsevier B.V. on behalf of King Saud University.

According to the light quantum theory, when the semiconductor receives a light quantum energy hʋ higher than the band gap, the photoelectron overcomes the escape work and escapes, producing free electrons in the conduction band and positive holes in the valence band (Figure 1) [93]. The electron produced in this process exhibits a strong reducing ability in the electron-donor reaction, and the produced holes exhibit an oxidizing ability in the electron-acceptor reaction. In the reduction process, the electron can undergo a molecular oxygen reaction with superoxide anions, and in the oxidation process, electrons in water or hydroxyl ions can be supplemented into holes to produce hydroxyl radicals. The superoxide anions and hydroxyl radicals produced in these two processes can degrade organic matter, and microorganisms and bacteria will be eliminated by OH^−^ and O_2_^−^ to complete the entire photocatalytic process [94,95,96]. The overall water decomposition reaction and the complete water decomposition reaction are as follows.

Overall water decomposition reaction:

(1) $$H_{2}O\overset{h\upsilon }{\to }\frac{1}{2}O_{2}+2H^{+}$$

(2) $$2H^{+}+2e^{-}\to H_{2}+\frac{1}{2}O_{2}$$

Complete water decomposition reaction:

(3) $$2H_{2}O\overset{h\upsilon }{\to }O_{2}+4H^{+}$$

(4) $$4H^{+}+4e^{-}\to 2H_{2}+O_{2}$$

During the catalytic splitting of water into H_2_, the CO_2_ that is generally reduced to CO can also be reduced to methane or ethane using special materials [97].

The photocatalytic reaction of metal oxides contains the above general photocatalytic reaction mechanism. The difference is that metal oxide photocatalysis mainly involves a catalyst containing transition metals. The whole cycle includes four main steps: oxidation, reduction, addition, and removal. The main mechanism is that the d-orbital electrons in the transition metal has the characteristics of easy separation or addition, which makes it easier to carry out redox reactions. At the same time, the requirement of d-orbital bonding and bond-angle matching is low and the bonding energy is not high, which is beneficial for the formation of low-orbital bonding of reactants. In some of the transition metal catalytic systems, after the introduction of platinum, palladium, rhodium, silver, and other precious metals, these transition metals can be used as photon acceptors to produce photoelectrons, which is conducive to the formation of active compounds, quantum light, and effective hole separation [98,99].

### 2.2. Graphene Optimization Method for the Photocatalytic Effect

#### 2.2.1. Graphene Matrix Composites and Graphene-Doped-Metal Matrix Composite

Graphene can be doped with B, Se, N, P, O, S, F, Cl, Br, I, etc. [100,101,102,103,104,105,106,107]. Titanium-based materials are widely used in purification and disinfection, building materials, and agriculture because of their non-toxicity, high reaction efficiency, and economy. They were considered to be the frontier materials in the field of photocatalysis in the previous stage [107,108]. TiO_2_-composite graphene nanomaterials have been proved to have better photoelectrochemical activity and photocatalytic activity [109,110,111,112]. The reason is that GO can fully act as an electron collection library to accept excited electrons from TiO_2_. At the same time, a Ti–O–C bond is formed that introduces the TiO_2_ bandgap intermediate state to promote a visible light reaction. Unfortunately, TiO_2_ also has the limitation of a light wave frequency. It is only suitable for the ultraviolet band, while ultraviolet only accounts for 4% of the solar spectrum, and a large number of electromagnetic waves in the band cannot be used [113]. In order to further develop photocatalytic energy efficiency and maximize the use of solar clean energy, various types of TiO_2_ photocatalytic derivatives of new materials have emerged ceaselessly and have been developed into a variety of composite types of metal oxides, organic photocatalytic semiconductors, etc. An organic photocatalytic semiconductor is a good alternative material. Its efficient photoelectric conversion efficiency and longer service life have a profound impact on solving the increasingly tense resource and environmental crisis and energy shortage. However, an unsatisfactory aspect is that there are fewer active sites in the material, the two carriers excited by the energy given by the light have a short interval, and the overall photocatalytic efficiency is not high, which make the development of this material still have great limitations [114,115].

In addition to metal-based materials, graphene-based composite conductive materials are also promising research directions. The main problem of this graphene-based material is that the free electrons and holes diffuse from the conduction band and valence band and then the free electrons and holes recombine quickly, losing the conductivity. This phenomenon affects photochemical stability and ultimately leads to a decrease in photocatalytic efficiency. The macroscopic performance shows that the material has high resistance. At present, there are a large number of strategies to solve the problem of the recombination of free electrons and holes excited by the optical quantum [116,117,118,119]. Now the more efficient solution for photocatalysis is based on the doping of graphene. The main reason for doping is that doping other elements can improve the absorption electromagnetic wave region of photocatalysis, change the graphene semiconductor from a broadband gap to a narrow band gap, reduce the bandgap energy, improve the photochemical stability, work efficiently in the absorption band, and further improve the photocatalytic efficiency [120]. Due to the special layered structure of graphene, it can carry and immobilize other materials, and it has obvious composite material characteristics, making the composite material composed of graphene and various semiconductors a new generation of more promising photocatalytic materials [121,122].

In addition to the above mentioned, graphene can be used as a photocatalytic material substrate and as a co-catalyst. In order to improve the photocatalytic effect of graphene materials, doped N has particularly prominent derivatives [80,123]. At the same time, nitrogen-doped (N-doped) graphene has been shown to be a high-performance co-catalyst that can effectively increase hydrogen production [124]. Liu et al. anchored Ni onto an N-doped graphene (NG) material as a co-catalyst for SrTiO(Al), effectively carrying the Ni-based material to improve the photocatalytic water splitting effect. In this experiment, the N on graphene can be used as the anchor for a single metal atom, and the atomic coupling of the single metal atom, Ni, is limited [125]. In this way, the effect of precious metals as co-catalysts can be achieved without the high-cost limits of precious metals. Liu’s team provided a good direction for photocatalytic water splitting, and in the past two years, Yang and Li’s team, advancing further in this direction, have proposed an N and S co-doped approach to obtain a nitrogen and sulfur co-doped graphene that has a better effect than single-doped N [126,127].

#### 2.2.2. Structure Design

Graphene’s ability to function as a photocatalytic substrate material and auxiliary material has a lot to do with its structure [128]. It is well known that monolayer nanomaterials are classified as two-dimensional structures, with graphene being the most common. Multilayer graphene stacked by chemical bonds is classified as a three-dimensional structure.

The enhanced catalytic effect of high-dimensional graphene can be attributed to the fact that, compared with one-dimensional graphene, two-dimensional and three-dimensional graphene can reduce the Coulomb binding energy of electron holes and increase the specific surface area to solve the shortcomings of the loose substrate contact and fewer active sites on graphene. At the same time, fixing metals at specific sites can also avoid the aggregation of metal oxides [129]. In particular, graphene materials with a three-dimensional porous structure have the synergistic effect of light absorption and size-stacking to form a higher specific surface area, which is conducive to multiple reflections of light and improves the light capture ability. They are also widely used in capacitors [130,131].

Graphitic Carbon Nitride (g-C_3_N_4_) is a two-dimensional material that has been intensively studied in ozone oxidation and in composites with rhodium phosphide [132]. Han reported a porous g-C_3_N_4_ material with heterostructural defects. They observed enhanced water-splitting hydrogen-production efficiency and photocatalytic H_2_-evolution activity by the heterojunction and the construction of defective ultrathin two-dimensional materials, respectively [133]. Liu et al. improved the structure of g-C_3_N_4_ and divided it into three groups: g-C_2.52_N_4_, g-C_1.95_N_4_ and g-C_1.85_N_4_. These three groups were also doped with Ag–Pd to form a two-dimensional composite material that strongly promoted photocatalysis. They controlled the ratio of carbon and nitrogen, and the removal rate of NO_2_^−^ and NO_3_^−^ was used as a reference for the degree of photocatalytic reaction. The XRD pattern showed that g-C_1.95_N_4_ had the best photocatalytic activity. After 3 h of reaction, the removal rate of NO_3_^−^ was 87.4%, and the removal rate of NO_2_^−^ was 61.8% [134]. This shows that by changing or utilizing the material structure of traditional photocatalytic materials, the catalytic hydrogen evolution performance can be significantly improved. At the same time, two-dimensional graphene semiconductor materials have lamellar stacking, and the intrinsic Dirac band structure and performance are not fully utilized. This is an obvious disadvantage of two-dimensional graphene, which is not mentioned in the above articles.

**Figure 2:**
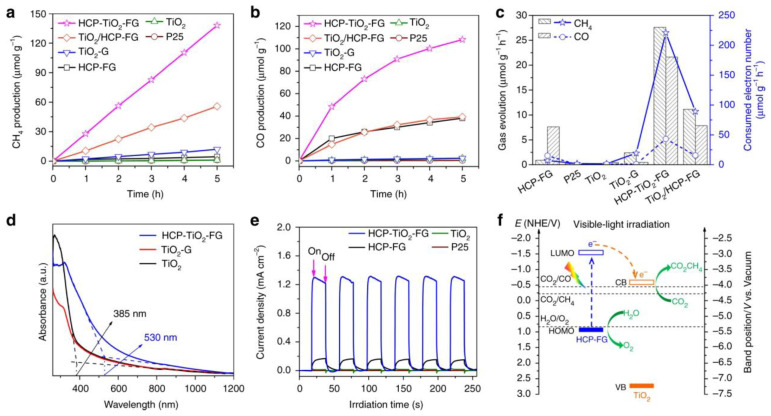
The photocatalytic performance of CO_2_ reduction, optical and photoelectrical properties, and mechanism of the charge transfer pathway. Time-dependent production of (**a**) CH_4_ and (**b**) CO in photocatalytic CO_2_ reduction with different catalysts under visible light (λ ≥ 420 nm). The photocatalytic reactions were carried out in a batch system under standard atmospheric pressure. The partial pressure of CO_2_ and H_2_O were constant, with the water content below the scaffold-loading photocatalyst. Under visible-light irradiation, the temperature of the water was measured to be about 50 °C. (**c**) Average efficiency of photocatalytic CO_2_ conversion with different catalysts during 5 h of visible-light (λ ≥ 420 nm) irradiation. (**d**) UV–Vis absorption spectra of TiO_2_, TiO_2_-G, and HCP–TiO_2_-FG catalysts. (**e**) Amperometric I−t curves of samples under visible-light (λ ≥ 420 nm) irradiation. (**f**) Proposed mechanism of charge separation and transfer within the HCP–TiO_2_-FG composite photocatalyst under visible-light (λ ≥ 420 nm) irradiation. Reprinted with permission from Ref. [135]. Copyright©2023 Springer Nature Limited.

The photocatalytic reaction of TiO_2_ composite photocatalytic materials is a good means to reduce the greenhouse effect gas, CO_2_. The efficiency of the photocatalytic conversion of CO_2_ into available chemical fuels mainly depends on the adsorption and diffusion of CO_2_ by the material. Wang et al. developed a porous composite structure by the in situ weaving of hyper-crosslinked polymers (HCPs) on TiO_2_-functionalized graphene (TiO_2_-FG) without adding precious metal co-catalysts. The porous HCP–TiO_2_ graphene composite structure was used as a photocatalytic material to achieve a CO_2_ absorption capacity of 12.87 wt% and a CH_4_ yield of 27.62 μmol g^−1^ h^−1^. The principle of effective CO_2_ absorption in this process is to introduce the material into the HCP layer (Figure 2).This method can increase the micropore volume and significantly increase the specific surface area of TiO_2_ graphene, thereby improving the overall energy efficiency of photocatalysis. This is a good example of a porous material application that offers a research direction for solar fuels [135].

Lee et al. provided a heterostructural treatment of graphite carbon three-dimensional nanomaterials. The team used hydrothermal treatment and the simultaneous reduction of GO and TiO_2_ crystals, and the absorption tail of GO-coated amorphous TiO_2_ was red-shifted (2.80 eV) [136]. Through hydrothermal treatment, during the conversion of TiO_2_ into amorphous TiO_2_, Ti atoms are rearranged, GO is reduced, and unpaired electrons are formed. The unpaired electrons and Ti atoms reduce the conduction-band level of TiO_2_, thereby reducing the band gap and improving the conductivity and photocatalytic effect. Qiu’s team provided a new idea for the treatment of graphene heterostructures by self-doping Ti^3+^ to place GO and TiO_2_ on the surface of graphene. GO-cracking into small size graphene; the smaller the size of the graphene, the more binding surface with TiO_2_ there is in the structure to promote the efficiency of photocatalysis. Moreover, this process also generates Ti–O–C bonds to form shallow surface defects, and the band gap is slightly reduced and red-shifted to 2.98 eV [137].

Many functional catalysts are used to enhance two-dimensional graphene phase carbonitrides [138,139,140,141,142]. The use of functional catalysts on three-dimensional porous graphene phase carbonitrides (g-C_3_N_4_/GO (p-CNG)) is also a good idea. Li’s team fixed Au, Pd, Pt, and other precious metals on the three-dimensional p-CNG skeleton. By monitoring the positive correlation between the metal content and the diffraction intensity of the three-dimensional porous-graphene-phase carbon nitride skeleton, it was determined that the three metals were successfully anchored on the three-dimensional porous-graphene-phase carbon nitride body. Subsequently, the performance, photostability and thermoelectrochemical performance of the hydrogen evolution reaction (HER) were tested. Because precious metals can be used as electron acceptor sites, the effect of electron-hole separation is significantly improved, and it has strong HER activity under simulated solar light (SSL). The test results show that the 3D p-CNG–Pt composite catalyst exhibits better HER activity than the 3D p-CNG–Au and 3D p-CNG–Pd composite catalysts under optimal conditions. This is due to the effective charge transfer of platinum and 3D p-CNG skeletons, which shows better HER kinetic curve linearity and lower catalyst dose consumption and results in better durability. In terms of thermoelectrochemistry, the 3D skeleton exhibits a wider absorption boundary (531 nm) than the 2D skeleton, and the anchored Au, Pd, and Pt significantly improve the photoreaction effect of the 3D p-CNG composite catalyst due to the improvement of the electron transition pathway [143].

Wang’s team provided a new approach to the photocatalytic charge transfer pathway. The team used the characteristics of the ordered structure of the metal organic layer to easily obtain active sites and prepared a 1.5 nm metal coordination layer with rGO as an electron mediator. The ultrathin two-dimensional metal–organic layers (MOLs) are distributed on the two-dimensional template constructed by rGO. The synergistic effect and electronic mediation of the two compensate for the gap between heterogeneous catalysts and homogeneous antenna molecules, improve the energy efficiency of photocatalysis and CO_2_ absorption, and significantly improve the activity of the photocatalytic reduction reaction [144].

**Figure 3:**
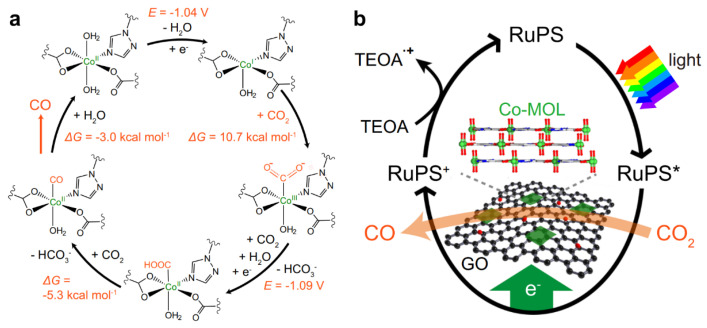
(**a**) Calculated mechanism with the molecular unit of Co−based metal−organic frameworks (MOFs) for photocatalytic CO_2_−to−CO conversion, showing the calculated redox potentials and free energy changes. (**b**) Proposed photocatalytic mechanism. Reprinted with permission from Ref. [145]. Copyright©2023 Springer Nature Limited.

Although metal-doped photocatalysts have been extensively studied, as described above, the efficiency and effectiveness of photocatalytic hydrogen production have been significantly improved. However, the use of these doped materials is still challenging in many aspects, especially with regard to their impact on the environment. For example, if the material is applied to too many metals, the cost of subsequent waste recycling will be increased. If not handled properly, this may lead to regional heavy metal pollution. This runs counter to the previous idea of improving global energy tensions and environmental crises through in-depth research on photocatalytic technology. Therefore, research on promoting the photocatalytic effect by relying on the characteristics of different dimensions of graphene-based materials is still advancing. Researchers want to improve the photocatalytic effect of graphene composites through the dimension of materials and the introduction of non-metallic elements, such as carbon, hydrogen, oxygen and phosphorus, to reduce the content of doped metals as much as possible, and even not introduce the whole process. Based on the photocatalytic process of perylene bisimide (PBI) supramolecular materials, the addition of zero-dimensional (or microscopic three-dimensional) graphene quantum dots (GQDs) significantly improve the effect of visible light photocatalysis (Figure 3). The small particle size of GQDs can be used to place high active sites and install PBI layer by layer through electrostatic interaction to form GQD/PBI supramolecular composites. The advantage of these GQD composites is that they can interact with the π-π of PBI to form long-range electron delocalization, and the quantum confinement effect also promotes the transfer of electrons from GQDs to PBI, which improves the reduction ability of PBI and the production characteristics of H_2_. This study undoubtedly provides ideas and future research directions for supramolecular organic photocatalysis at the level of quantum modification [145].

**Figure 4:**
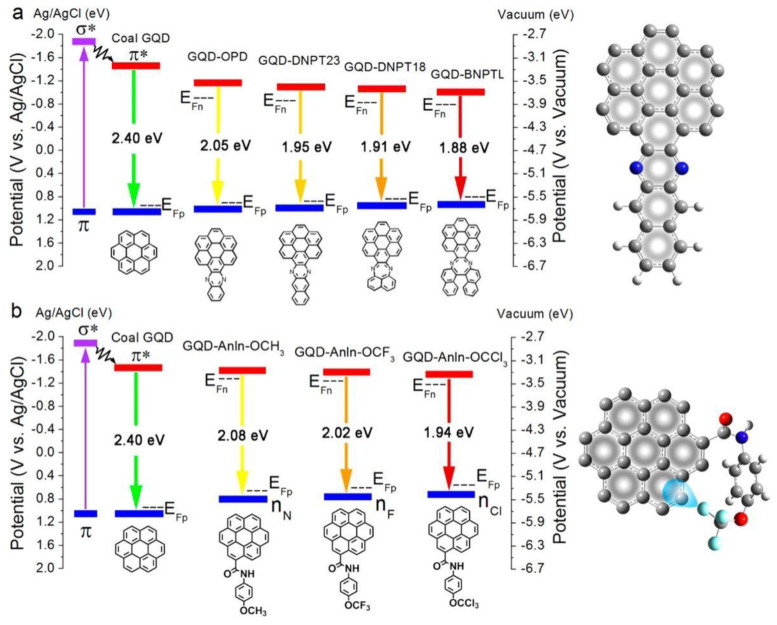
Illustration of energy level diagram and simplified structure of (**a**) coal GQD, GQD−OPD (o−phenylenediamine), GQD−DNPT23, GQD−DNPT18, and GQD−BNPTL and (**b**) coal GQD, GQD−Anln−OCH_3_, GQD−Anln−OCF_3_, and GQD−Anln−OCCl_3_. The Fermi levels for p-type conductivity (E_Fp_) and n−type conductivity (E_Fn_) are indicated in the energy diagram. Reprinted with permission from Ref. [146]. Copyright©2023 American Chemical Society.

**Figure 5:**
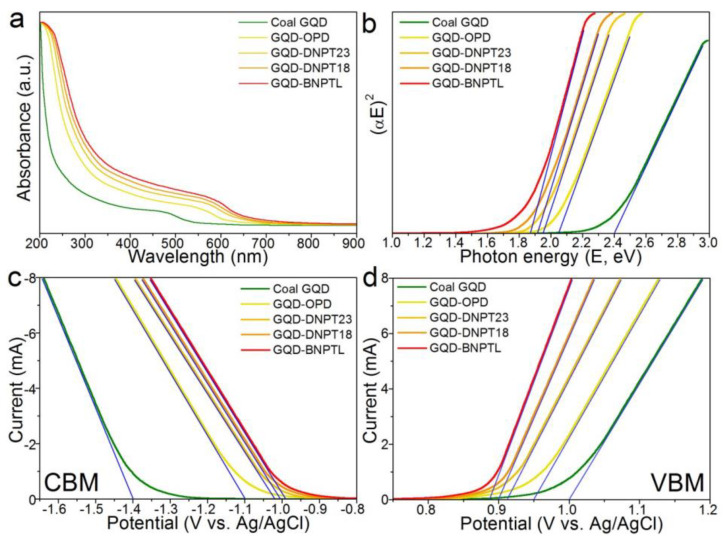
UV–Vis absorbance spectra (**a**) and accordingly obtained plots (**b**) of (αE)2 versus photon energy (E) (where α denotes the absorbance coefficient) of coal GQD, GQD-OPD, GQD-DNPT23, GQD-DNPT18, and GQD-BNPTL. The horizontal intercept of the tangent line in (**b**) indicates the bandgap of each GQD type. (**c**) Cathodic linear sweep voltammetry (**c**) and anodic linear sweep voltammetry (**d**) of coal GQD, GQD-OPD, GQD-DNPT23, GQD-DNPT18, and GQD-BNPTL. The horizontal intercept of the tangent line in (**c**) or (**d**) determines the conduction band minimum (CBM) or the valence band maximum (VBM) of each GQD type, respectively. Reprinted with permission from Ref. [146].Copyright©2023 American Chemical Society.

The Yan team studied the reduction process of functionalized GQDs to decompose water and absorb CO_2_ under visible light. They reported two ways to narrow the band gap of GQD and explained the mechanism. GQD-Anln-OCH_3_, GQD-Anln-OCF_3_ and GQD-Anln-OCCl_3_ have a Z-scheme structure, and charge separation can promote coupled photocatalysis (Figure 4). The absorption range of all GQDs is limited to 200–300 nm due to the π-π transition of the sp^2^ substrate. Yan also compared the optical absorption capacities of GQD derivatives: GQD-BNPTL > GQD-DNPT18 > GQD-DNPT23 > GQD-OPD > GQD (Figure 5) [146].

### 2.3. Graphene Electrocatalysis

The high specific surface area of graphene can provide more active sites and electron transport channels, which can increase the contact area of reactants and catalysts and the electron transfer between catalysts and electrodes, thereby increasing the catalytic effect [147,148,149,150]. At the same time, graphene can also act as a carrier for a stable catalyst. Fixing the catalyst on graphene can improve the dispersion and stability of the catalyst [151,152,153,154,155]. In addition, the functional groups carried on the surface of graphene can also provide additional active sites to regulate the catalytic object or catalytic efficiency. The electrocatalytic mechanism of graphene is based on its high surface area and conductivity, electron transfer ability, catalyst support, and surface functional groups. These factors work together to significantly improve the efficiency and performance of catalytic reactions [74,156,157,158,159]. The oxygen reduction reaction (ORR) and the oxygen evolution reaction (OER) are crucial for bifunctional electrocatalysts, which are often used in practical applications of rechargeable metal–air batteries [160].

**Figure 6:**
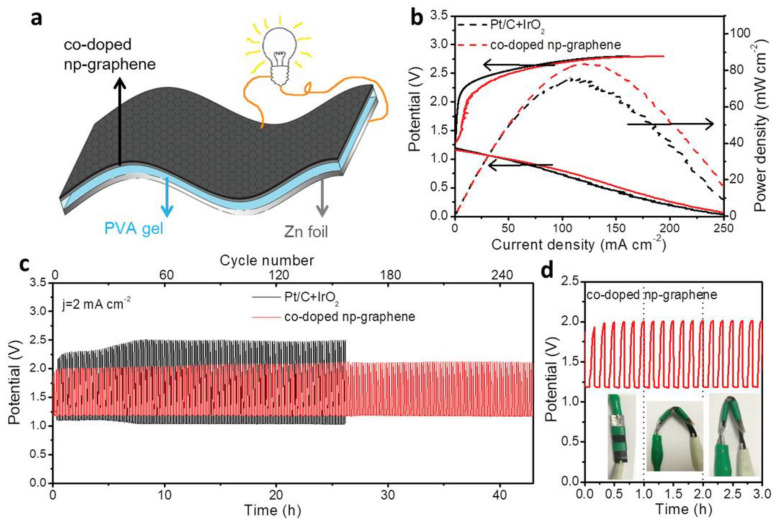
(**a**) Schematic diagram of the co−doped np-graphene−based all−solid-state Zn−air battery. (**b**) Polarization and power density curves of the batteries. (**c**) Discharge/charge cycling curves at 2 mA cm^−2^ and (**d**) discharge/charge curves under different bending states. Reprinted with permission from Ref. [78]. Copyright© John Wiley & Sons, Inc. All rights reserved.

A 3D nanoporous graphene (np-graphene) bifunctional electrocatalyst is formed by the nitrogen doping and nickel doping of 3D nanoporous graphene, in which nitrogen and nickel have two forms: single atom and cluster. A rechargeable all-solid-state zinc–air battery was prepared by using nitrogen and nickel co-doped np-graphene as a self-made flexible air cathode, PVA gel as an electrolyte, and Zn foil as an anode. As shown in Figure 6 below, the open circuit voltage of this air battery is 1.35 V, and the maximum power density of discharge polarization is 83.8 mW cm^−2^. Moreover, there is only a slight performance loss after 258 cycles, and the bending of the battery at different angles does not affect its performance, showing good cycle stability and flexibility [78].

The single atoms of nickel and iron are respectively embedded in the inner and outer walls of graphene hollow nanospheres (GHSs) to form a new type of highly active Ni-N_4_ and Fe-N_4_ Janus structure with distributed self-assembly, in which a planar configuration is formed to coordinate with four nitrogen atoms. The Janus structure on the inner and outer walls of the GHSs separates the ORR and OER active sites. The outer Fe-N_4_ site plays a major role in the ORR activity, and the inner Fe-N_4_ site plays a major role in the OER. When Ni-N_4_/GHSs/Fe-N_4_ is used as the air cathode, the specific capacity of Ni-N_4/_GHSs/Fe-N4 can reach 777.6 mAh gZn^−1^, and the energy density can reach 970.4 Wh kgZn^−1^, showing excellent bifunctional electrocatalytic activity. The Ni-N_4_/GHSs/Fe-N4-based zinc–air battery still maintains stable electrocatalytic performance after about 200 h of operation, which is considered excellent cycle stability [161].

## 3. Pollutant Degradation

### 3.1. Removal of Typical Pollutants

#### 3.1.1. Antibiotics

In recent years, antibiotics have been widely used in human treatment, animal husbandry, and aquaculture [162]. However, antibiotics do not have a good metabolic effect on organisms. The widespread use of antibiotics has caused antibiotic residues to flow into the environment along with raw materials, causing ecological damage. If humans drink water containing antibiotics for a long time, it will also cause damage to human health [163]. Nowadays, adsorption or catalytic oxidation methods, such as the use of natural zeolite, bentonite, activated carbon, carbon nanotubes, biochar, etc., are usually used to remove antibiotics from the water environment [164]. Membrane filtration, oxidation, photocatalysis, and biodegradation can also be used for antibiotic removal [165]. The scientific community has been researching the removal of antibiotics by various nanomaterials such as graphene for a long time [166]. The Ibrahim team studied a new type of on-line potential monitoring technology for antibiotics. It can effectively monitor the degradation or elimination of antibiotics [167].

Antibiotics have a negative impact on plants, aquatic organisms, and microbial communities, causing damage to the ecological environment and affecting human health. The common antibiotics contained in wastewater are tetracycline antibiotics, quinolone antibiotics, β-lactam antibiotics, macrolide antibiotics, sulfonamide antibiotics, etc. [162]. As one of the broad-spectrum tetracycline antibiotics, oxytetracycline (OTC) was identified as having small side effects and was thus widely used in the 1960s and 1970s. However, as OTC became widely used in livestock and aquaculture, people gradually discovered that organisms could not fully absorb these antibiotics. About 90% of OTC enters into the ecological environment [168], which leads to OTC accumulation in the aquatic environment; over time, this will produce resistant bacteria and resistance genes, greatly reducing the effective use of these antibiotics [169]. Another antibiotic, ofloxacin (OFX), one of the third-generation quinolone antibiotics, has been used unrestrainedly and discharged at will. Five years ago, the concentration of antibiotics in groundwater/surface water around the world reached 30 mg/L, which is concerning data [170].

It has been found that the removal efficiency and kinetic constant of rGO-Fe_3_O_4_ composites formed by adding GO are further improved compared with using Fe_3_O_4_ alone. The OFX removal rate can even reach 99.9% complete removal. Compared with the alkaline environment, the degradation rate and ability of OFX is more effective under acidic conditions [171]. After 0.5 min visible light irradiation, the degradation rate of ciprofloxacin hydrochloride (CIP) by the perylenetetracarboxylic diimide (PDI)/rGO composite film can reach 94.31% (10 mg/L). This composite film has been found to have two functions: photocatalytic degradation and photothermal conversion. By using rGO as an additive material, the optical absorption range of nano-PDI powder self-assembled using hydrochloric acid can be extended to the near-infrared band. Coupled with the selectivity of the upper band gap, the ions and pores of the upper nano-PDI will relax to the edge of the band, so the excess energy can be converted into heat. The practical significance of studying this PDI/rGO composite membrane shows that there is a visible-light-responsive graphene-based photothermal catalytic material that can achieve two functions. It can degrade antibiotics while recovering pure water in actual water samples to achieve the purification and recovery of wastewater [172].

Using citric acid-modified GO and carboxymethyl cellulose membrane (GO-CMC) to remove antibiotics, the adsorption capacity of OTC, quinic acid (OA), and trimethoprim (TMP) can reach 102.05, 252.68 and 370.93 mg/L, respectively. Antibiotics are deposited on the surface of GO by π-π interactions and cation-π bonds. The citric acid-modified GO-CMC membrane can be reused and maintains stable recyclability after 5 times of recycling, which can remove antibiotics in wastewater [173]. For promoting the degradation of OTC in water, a graphene–TiO_2_–Fe_3_O_4_ nanocomposite plasma has a good degradation effect. Compared with rGO–TiO_2_, the addition of Fe_3_O_4_ enlarged the specific surface area of the composites, accelerated the separation of electron-hole pairs, and increased the magnetic strength, which made the catalyst easy to separate from water. At the best doping amount of Fe_3_O_4_ of 20 wt%, the removal efficiency can reach 98.1%, which is the highest removal efficiency. Other influencing factors include the catalyst dosage, air-flow rate, peak voltage, and pH value, and their optimal values are 0.24 g/L, 4L/min, 18, and 3.2 kV, respectively. When the above conditions are reached, rGO–TiO_2_–Fe_3_O_4_ has the best removal performance. The rGO–TiO_2_–Fe_3_O_4_ still has high catalytic performance after four uses [174].

Heterogeneous photocatalytic technology based on the TiO_2_-based catalytic system is widely considered to remove pollutants in the environment without producing secondary pollutants, which is conducive to environmental protection [175]. For cephalosporin antibiotics, the photocatalytic activity of TiO_2_/N-doped porous graphene nanocomposites (TiO_2_/NHG) is better than that of bare p25 TiO_2_, TiO_2_/GO and TiO_2_/porous graphene frameworks. The results showed that the oxidative degradation rate of TiO_2_/NHG was affected by the catalyst loading, the initial antibiotic concentration, and the presence of H_2_O_2_. The complete mineralization of 25 mg/L of antibiotics was observed within 90 min of irradiation, and the activity level of the TiO_2_/NHG catalyst did not decrease significantly within three repeated cycles. The complete mineralization of antibiotics can be achieved only by sunlight irradiation within a reasonable time span, which means that the oxidative degradation of antibiotics remains efficient even in the absence of H_2_O [176].

Magnetic GO/ZnO nanocomposite (MZ) materials offer an excellent adsorption capacity with reusability for tetracycline antibiotics (TCs) in wastewater. The results show that the maximum adsorption capacity of MZ materials for TCs can reach 1590.28 mg g^−1^ at pH = 6.0. At the same time, after four absorption cycles, the adsorption capacity of MZs still did not decrease significantly and maintained a relatively stable adsorption activity. MZ materials have been proved to have the advantages of fast separation speed, strong adsorption capacity, reusability, and simple operation in use [177]. Based on the raw material MXene Ti_3_C_2_, a graphene-layer anchored TiO_2_/g−C_3_N_4_ (GTOCN) photocatalyst was formed by a one-step in situ calcification method. This method of synthesis can not only act on antibiotics but also on another persistent organic pollutant, namely dyes. Under visible light, such as sunlight in daily environments, GTOCN will have highly oxidizing active ·O_2_ and ·OH, hence offering high-efficiency degradation of tetracycline (TC) and ciprofloxacin (CIP) antibiotics and bisphenol A (BPA) and rhodamine B (RhB) dyes [178].

The synergistic catalytic removal of thiamethoxam (TAP) induced by pulsed discharge plasma (PDP) was studied by using graphene–WO_3_–Fe_3_O_4_ nanocomposites. Compared with single WO_3_ and rGO–WO_3_ without Fe_3_O_4_, rGO–WO_3_–Fe_3_O_4_ has a larger specific surface area and higher transfer rate of photogenerated carriers. Fe_3_O_4_ doping does not make the higher the better. With the gradual increase from zero, the TAP removal curve first increased and then decreased. When the Fe_3_O_4_ doping amount was 24 wt%, the catalyst dosage of 0.23 g/L obtained the best catalytic performance, and the removal rate of TAP could reach 99.3%. Acidic conditions and the presence of O_3_, H_2_O_2_, and ·OH are more conducive to the catalytic degradation of TAP [179].

The three-dimensional (3D) graphene can be designed as an anode to create an enhanced electron and mass-transfer photocatalytic circulation system, which can be used to remove ampicillin in wastewater and to enhance the antibacterial properties of water. It enhances electrons by using a three-dimensional graphene photoelectrode as an anode, a Pt/C air breathing electrode as a cathode, and C_3_N_4_-MoS_2_ loading. Perhaps due to the cleavage of functional groups such as amide bonds and peptide bonds, the removal rate of ampicillin by the photoanode reached 74.6% after the system was fully reacted in sewage for 2 h [180].

The 3D-MoS_2_ sponge modified by molybdenum disulfide nanospheres and GO adsorbs organic molecules and provides a multidimensional electron transport path, which has a positive effect on the degradation of advanced oxidation processes (AOPs), especially for aromatic organics. After pilot-scale experiments in 140 L wastewater, it still maintains efficient and stable activity for AOPs. Even after 16 days of continuous experiments, 3D-MoS_2_ can still maintain a degradation rate of 97.87% in wastewater containing 120 mg/L antibiotics. This is of great practical and economic interest for industrial applications; if the sponge could be produced industrially in large quantities, the cost of treating a ton of wastewater would be only USD 0.33 in the future [181].

**Figure 7:**
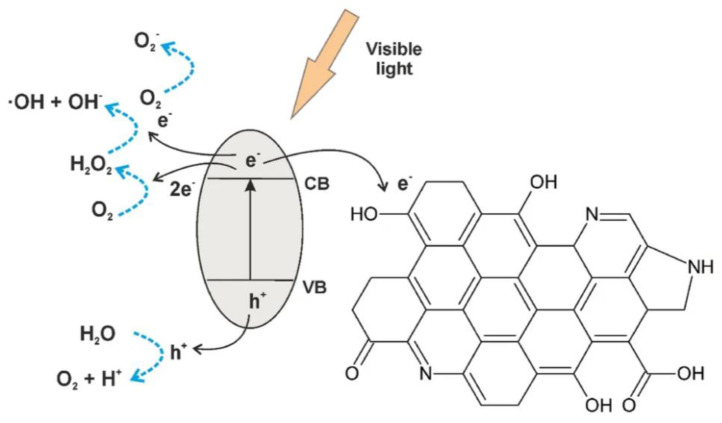
Representation of photon absorption, electron transfer, and generation of reactive species under visible−light irradiation of a Fe oxide/Fe hydroxide/N−rGO nanocomposite. Reprinted with permission from Ref. [182].Copyright©2023 Springer Nature Limited.

Ion-doped GO nanocomposites can perform photon absorption, electron transfer, and the generation of active species under visible-light irradiation. For example, an iron oxide/hydroxide N-doped graphene-like nanocomposite has been synthesized by a laser-based method to remove antibiotics from wastewater. The method is shown in Figure 7. Moreover, this nanocomposite is still environmentally friendly during the preparation process [182].

#### 3.1.2. Dye

One of the main causes of surface water pollution is from the textile industry, where waste from the printing and dyeing process is directly discharged into the water environment without qualified treatment [183].The increasing concentration of dyes in the water environment seriously affects the refractive index of light, which limits the growth of aquatic plants and further negatively affects the self-purification of the ecosystem. More water resources in barren water resources are no longer suitable for organisms, let alone for household and industrial use [184].

**Figure 8:**
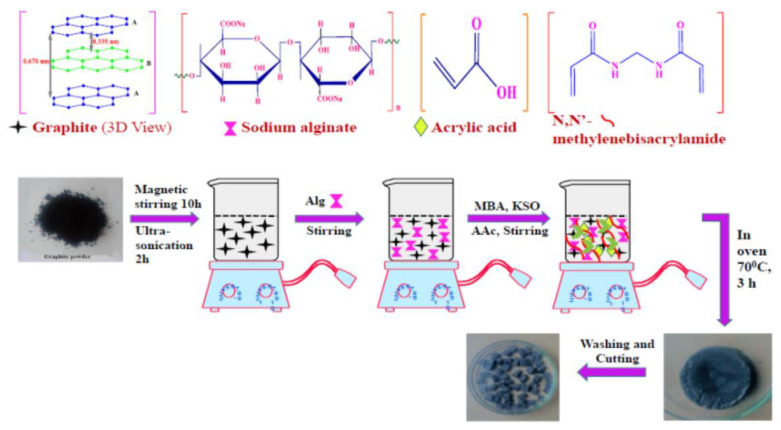
Preparation route diagram of hydrogel composites. Reprinted with permission from Ref. [187]. Copyright©2020 Elsevier B.V. All rights reserved.

A triphenylmethane cationic dye—Malachite green (MG)—is a common dye in the dyeing process of the textile industry. However, due to the presence of nitrogen (N_2_), MG consumption may lead to many serious human health issues, e.g., cancer, etc. [185]. Ozone oxidation, oxidation, membrane filtration, flocculation, biosorption, and electrochemical methods are often used to remove MG dyes on the market. However, these methods are not only costly, but they also have requirements for reaction conditions, such as dissolved oxygen demand. Moreover, due to the problems of high sludge production, short half-life and slow process, the removal efficiency of dyes is not high [186]. As shown in Figure 8, the porous sodium alginate/graphite-based composite hydrogel was modified by the grafting polymerization of acrylic acid on sodium alginate, and graphite powder was loaded to enhance its adsorption capacity. In terms of the effective adsorption for organic pollutants, the maximum adsorption capacity for malachite green dye can reach 628.93 mg g^−1^. The hydrogel complex showed sustainable usability and could still adsorb 91% of MG after three consecutive dye adsorption–desorption cycles [187].

A novel 3D magnetic bacterial cellulose-nanofiber/GO-polymer aerogel (MBCNF/GOPA) mesoporous structure with a high surface area of 214.75 m^2^g^−1^ can be used to remove malachite green (MG) dye from aqueous solution with a maximum adsorption capacity of 270.27 mg g^−1^.When the reaction environment meets the conditions of temperature (25 °C and contact with 30 mg/L MG concentration solution for 25 min, the solution with pH = 7.0 and 5 mg MBCNF/GOPA can have the best performance. The adsorption efficiency of MBCNF/GOPA remained above 62% after 8 times of recovery using 0.1 mol/L acetic acid/methanol in a 1:2 mixing ratio [188].

A polyvinylidene fluoride (PVDF)–polyaniline (PANI) and GO mixed membrane was prepared by incorporating PANI–GO as a nano-filler, which greatly improved the antifouling performance and solvent content. The pure water flux through the membrane increased from 112 to 454 mL/m^2^·h. Compared with other compositions and doping materials, the nanocomposite membrane with 0.1% *w*/*v* GO is superior. When operating conditions reach 0.1 MPa operating pressure, the dye rejection rate can reach 98%. After several tests on the membrane, the flux recovery rate of almost all dyes can be stabilized at 94%, while the removal rate of individual dyes such as methyl orange can reach 95%, and that of Allura red can be as high as 98% [189].

A nanocomposite hydrogel (NCH) formed by chitosan (CS) and carboxymethyl cellulose (CMC) crosslinked-modified GO has a significant adsorption effect on methylene blue (MB) and methyl orange (MO). At pH 7, the adsorption rate of 0.4 g/L CS/CMC-NCH for 50 mg/L MB was about 99%. At pH 3, the adsorption rate of 0.6 g/L CS/CMC-NCH for MO was about 82%. The adsorption capacity of CS/CMC-NCH for MO is 404.52 mgdye/gads, and the adsorption capacity for MB can reach 655.98 mgdye/gads. More importantly, the composite hydrogel has a stable adsorption performance for the dye after continuous use of 20 adsorption–desorption cycles. CS/CMC-NCH also has an excellent effect on the removal of anionic and cationic dyes [190]. The novel lysine and ethylenediamine double-crosslinked graphene aerogel (LEGA) exhibited a 3D interconnected porous structure, which greatly increases the adsorption capacity for the MB dye. Compared with other substrate materials, the compression performance of LEGA was significantly improved after adding lysine, and the adsorption capacity of MB could reach 332.23 mg/g [191].

For the removal of the crystal violet (CV) dye, it was found that two kinds of nanocomposite hydrogels, acrylamide-bonded sodium alginate (AM-SA) and acrylamide/GO sodium alginate (AM-GO-SA), can be synthesized by the free radical method, and both of them have good adsorption properties for CV. Compared with other influencing factors, the removal efficiency of AM-SA and AM-GO-SA was more dependent on pH, and the maximum single-layer adsorption capacity could reach 62.07 mg/g and 100.3 mg/g [192].

A novel GO/poly (N-isopropylacrylamide) (GO/PNIPAM) composite system removes organic dyes in water by a similar extraction mechanism and undergoes a reversible sol–gel transition at a temperature higher than the lower critical solution temperature. PNIPAM is anchored on the surface of GO to prevent the reduction of GO and inhibit its aggregation, which greatly improves the stability of GO dispersions. Moreover, the dye can be effectively adsorbed and enriched in the gel phase, which is convenient for its separation from water during the extraction process [193].

#### 3.1.3. Oil and Organic Solvents

The rapid development of modern transportation, petrochemical, and marine engineering has accelerated the release of a large amount of oil into the sea and rivers, causing energy loss, destroying the local ecological environment, and seriously endangering the sustainable development of society [194].The urgent need to solve the problem of oil pollution is self-evident. The public has focused on oil/water separation technology and its ability to treat industrial oily wastewater and oil spill accidents. However, most of the traditional oil/water separation materials are based on activated carbon, polypropylene sponge, and zeolite, and other microporous structure absorbents have the disadvantages of limited absorption capacity and poor wear resistance [195].

Based on polyimide (PI), a novel zeolitic imidazolate framework-8/thiolated graphene (ZIF-8/GSH) nanofiber membrane can be prepared by electrospinning and in situ hydrothermal synthesis. The membrane has superhydrophobicity/superoleophilicity and can effectively purify oily wastewater. For various oil/water mixtures and water-in-oil emulsions, the separation efficiency of oil and water can reach 99.9% through the action of fiber membranes. More importantly, the film maintains superhydrophobicity without requiring the environment to remain under harsh conditions. Under harsh reaction conditions, such as excessive acid and alkali, long-term contact with salt and corrosive organic solvents, high temperature irradiation, mechanical wear, ultrasonic treatment and other simulated environmental conditions, ZIF-8 @ GSH can still exhibit excellent photocatalytic degradation efficiency. This shows that the membrane has self-cleaning and active antibacterial abilities and can maintain its performance through the mechanical and chemical environment, which makes future industrial applications very promising [196].

With the release of a large amount of oil pollution into the water environment, the emergence of reusable superhydrophobic oil adsorption materials are required. A magnetic superhydrophobic polyurethane sponge (Fe_3_O_4_/OA/GO-PU) was formed based on a 3D microstructure by linking GO and coating with functionalized oleic acid Fe_3_O_4_ nanoparticles. It can be water repellent, with a contact angle of 158°, and has high selectivity in contact with organic solutions and oils. Theoretically, the microstructure polyurethane (M-PU) sponge has a capacity of 80–160 g/g, and it can undergo 15 adsorption cycles. The oil can be selectively extracted from the wastewater, exhibiting excellent recyclability and the ability to be recovered in a static state using an external magnetic field. Compared with the absence of a magnetic field, the increase of M-PU adsorption capacity seems to be driven by magnetic field exposure due to the enhancement of surface-active sites of the M-PU sponge. The continuous collection of kerosene from the surface water while cleaning wastewater with Fe_3_O_4_ @ OA @ GO-PU is cost-effective, highly selective, and an excellent recyclable, environmentally friendly oil-spill cleanup option [197].

**Figure 9:**
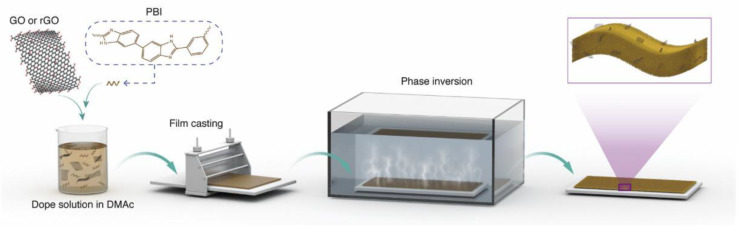
Schematic representation of the membrane fabrication steps. Reprinted with permission from Ref. [198]. Copyright©2020 The Authors. Published by Elsevier B.V.

Using phase inversion technology and the dip coating method, a nanocomposite film was composed of polybenzimidazole (PBI), graGO, rGO, and polydopamine (PDA) in a coated and uncoated manner as shown in Figure 9. When 0.5–1.5 wt% GO was added to the polymer matrix, the antifouling performance of the composite membrane could be improved to the highest extent, and the maximum flux of the membrane reached 91 L/m^2^·h·bar. Compared with the original single PBI material, the permeability of the water–oil mixture of the nanocomposite membrane increased by 17%, and the oil removal efficiency also increased from 80% to 100%. After four clean filtration cycles, the water-flux recovery rate (FRR) remained above 90%. Even without any alkaline and acidic cleaning, it exhibits good separation rate for oil-in-water emulsions and stable antifouling and antibacterial properties [198].

**Figure 10:**
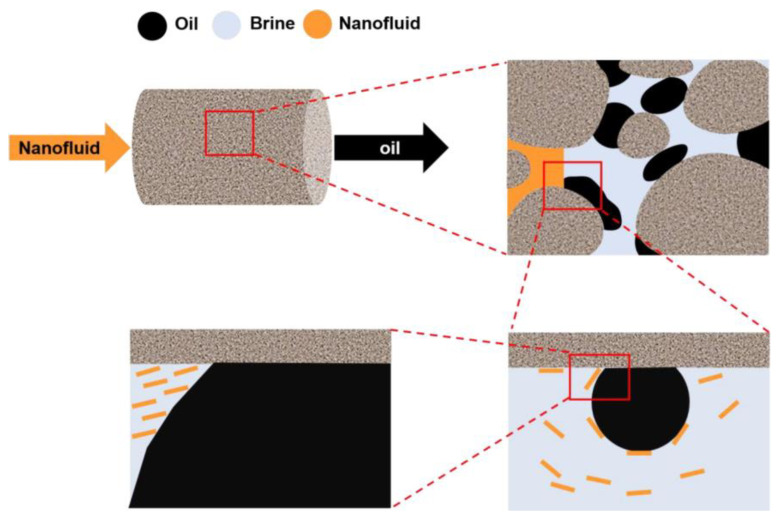
The schematic of displacement mechanism of P-GO-O. Reprinted with permission from Ref. [199]. Copyright© 2021 Elsevier Ltd. All rights reserved.

A novel poly (oxyethylene) graphene oxide-based nanofluid (P-GO-O) prepared as shown in the Figure 10 shows high temperature resistance and high salt tolerance in deionized water, with a potential of 39 mV. The recovery rate of octadecyl-aminated graphene oxide (GO-O) is only 6.7%, while that of P-GO-O is 17.2%, indicating that P-GO-O can improve oil recovery. This is due to the fact that its structural oil–water interfacial tension can be reduced to 12.2 mN/m under the action of P-GO-O, and the oil-wet surface is turned into a water-wet surface. Even under harsh conditions, P-GO-O still exhibits stable properties [199].

**Figure 11:**
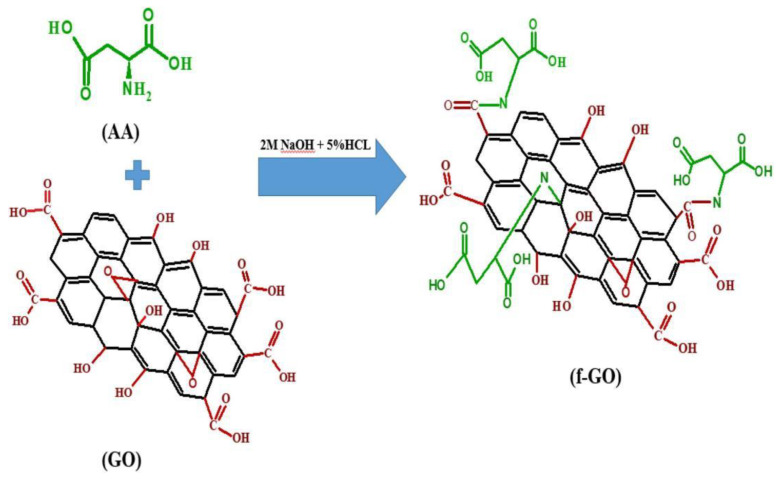
Functionalization of GO with aspartic acid. Reprinted with permission from Ref. [200]. Copyright©2020 The Author(s). Published by Elsevier Ltd.

As shown in Figure 11, a polysulfone (PS) mixed-matrix membrane containing aspartic acid (AA)-functionalized graphene oxide (fGO) has good hydrophilicity, water permeability and oil repellency at a very low GO loading. Thanks to the fluorine GO load, the fluorine GO load performance of the film is much higher than that without addition. The functionalization of AA introduces carboxyl and amino groups, which is beneficial to the performance improvement of the matrix membrane and improves the hydrophilicity and fouling removal rate. After adding very low concentrations of fGO, the incorporation of fGO in the PS membrane had a positive impact on the mechanical properties and antifouling properties of the membrane and enhanced the separation rate of the oil–water emulsion. The affinity of BSA for the membrane surface decreased, which means that the flux recovery of the fGO membrane after bovine serum albumin (BSA) contamination was higher. Compared with the monotonous original membrane, the water permeability of the composite membrane doped with 0.2 wt% fGO increased by 97% and the oil rejection rate reached 97.9% when the 200 mL oil emulsion was filtered [200].

**Figure 12:**
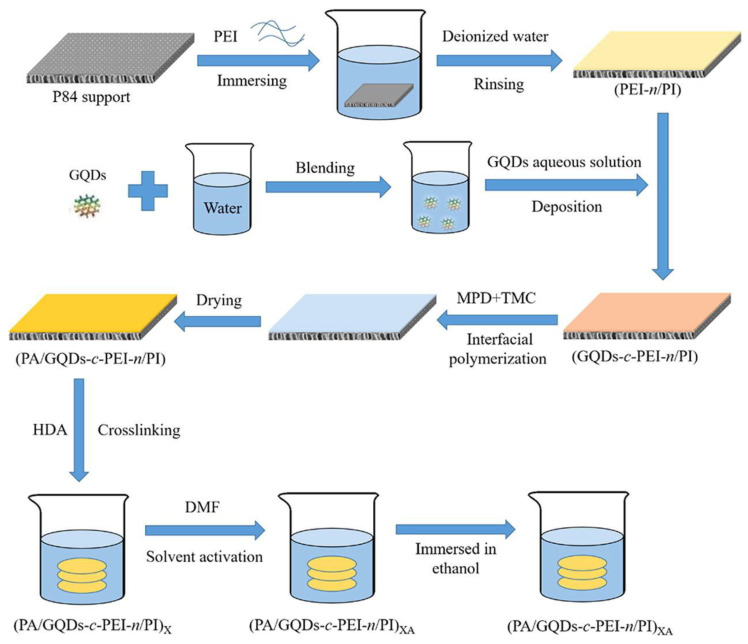
The fabrication process of the GQD-interlayered TFN-OSN membranes. Reprinted with permission from Ref. [201]. Copyright© 2019 Elsevier B.V. All rights reserved.

A small sheet of graphene oxide (SFGO) film for high-performance organic solvent nanofiltration (OSN) applications, using La^3+^ as a crosslinking agent and a spacer layer for insertion; moreover, it stabilizes the SFGO film selection layer and achieves selective molecular transport. The permeability of methanol is 2.9 times higher than that of large-flake graphene oxide (LFGO), and it has high selectivity for three organic dyes. More importantly, the SFGO-La^3+^ film exhibits at least 24 h of stable stability under hydrodynamic stress, which represents real OSN operating conditions. Through the interfacial polymerization (IP) of low-concentration resorcinol on the surface of the graphene quantum dot (GQD)-polyethylenimine (PEI)-modified polyimide substrate, a new nanocomposite (TFN) organic solvent nanofiltration membrane with a sandwich structure was prepared as shown in the Figure 12.The thickness of the IP skin layer of the GQD-interlayer OSN film is about 25 nm, and the average surface roughness is generally less than 2 nm, resulting in an increase in the penetration content of Harnol from 33.5 to 40.3 Lm^−2^h^−1^MPa^−1^, and the penetration rate of Rhodamine B from 87.4% to 98.7%. After long-term immersion in pure N, N-dimethylformamide (DMF), it showed superior solubility. After 81 days of storage at room temperature, it was stored at 80 °C for 45 days and then filtered with Bengal Rose (1017 Da) DMF solution at 25 °C for 5 days. There was no scar solute rejection, which proved the antifouling performance during long-term filtration [201].

### 3.2. Adsorption of Heavy Metals in Sewage

Common heavy metals in sewage, such as Cd, Zn, Pb, Fe, Cu, Hg, Ni, Mn, Co, etc., generally exist in trace amounts. Even in very small amounts, heavy metals are considered to be the most harmful, toxic and most widely distributed components in wastewater due to the mobility of the ions [202].The discharge of heavy metals into the water environment will not only adversely affect the ecological environment but also accumulate in soft tissues after entering the human body, seriously endangering human health and even threatening life and health [203]. For example, copper can lead to liver damage, insufficient blood supply, and night-time insomnia. It can also inhibit the activity of enzymes in the soil and affect the circular development of the ecological environment. Chromium can cause dizziness, headache, nausea, and diarrhea. Excessive inhalation of lead may lead to muscle spasm, even renal failure, and damage to the brain of infants. Mercury can cause rheumatoid arthritis and even threaten the normal work of the human nervous system circulatory system [204]. Due to heavy-metal ions being highly soluble, stable, non-biodegradable, and able to migrate in aqueous media, metal-contaminated wastewater can also cause harmful effects in plants, such as photosynthesis inhibition, and the reduction of seed germination rate, enzyme activity and chlorophyll synthesis [205].

In the past, traditional technologies were used, such as non-destructive processes using resins or adsorbents [206], non-destructive separation using semi-permeable membranes, and solvent separation techniques, which often have disadvantages such as low efficiency, insufficient removal, strict operating conditions, and high prices. Nowadays, high porous nanostructures such as graphene can be used as alternatives to remove heavy metals from contaminated water, e.g., graphite oxide (GO) for Pb^2+^ and Cd^2+^ has excellent adsorption capacity with great potential [134]. Because of its small particle size, large specific surface area and high adsorption efficiency, graphene can break through the limitations of conventional adsorbents and has become a good choice for removing heavy metal ions from water [207].

The preparation of a Bi_2_S_3_-BiVO_4_ graphene aerogel (SVGA) requires only a simple hydrothermal method, which can provide effective assistance in both the photogenerated electron transfer and photocatalytic ability of SVGA. The results show that removal rates of Cr (VI) and bisphenol A (BPA) using SVGA materials can be infinitely close to 100% after 40 min of adsorption and 120 min of photocatalysis under visible-light irradiation at 420 nm. The harmful Cr (VI) is preserved as low-toxic Cr (III) after photocatalysis on SVGA, and BPA is degraded into CO_2_ and H_2_O [208].

Due to the presence of oxygen-containing functional groups and high porosity, 3D magnetic fungal mycelia/graphene oxide nanofibers (MFHGs) can remove Co (II) and Ni (II) from high-salinity aqueous solutions, increasing the ion removal rate. The optimum reaction conditions were as follows: at 323 K and pH = 6.0, MFHGs could remove 97.44 and 104.34 mg/g of Ni (II) and Co (II), respectively, from 2 g/L Na_2_SO_4_ aqueous solution. Reductive self-assembly (RSA), one of the main materials of MFHGs, is cheap and environmentally friendly, so the cost of MFHGs is not high. They have excellent magnetization and large coercivity and can work normally in high-salinity water [209].

Under aerobic conditions, a novel graphene-like biochar supported trivalent iron (GB/nZVI) to remove Cd (II) and As (III). The main principle of removing As (III) is through oxidation and surface complexation, while the removal of Cd (II) mainly depends on surface complexation. At the same time, the strong synergy between GB and nZVI has a positive effect. The removal ability of GB/nZVI composites was significantly higher than that of pure GB and nZVI under both acidic and neutral conditions. The presence of As (II) significantly promoted the removal of Cd (III) when both ions were present in the same water environment. The maximum removal of As (III) can reach 181.5 mg/g when nZVI is 363 mg/g. The maximum removal capacity of Cd (II) can reach 46.4 mg/g when n ZVI is 92.8 mg/g. It is worth mentioning that the presence of phosphate and humic acid in the coexisting background ions has a reverse inhibitory effect on the removal of Cd (II) and As (III) [210].

**Figure 13:**
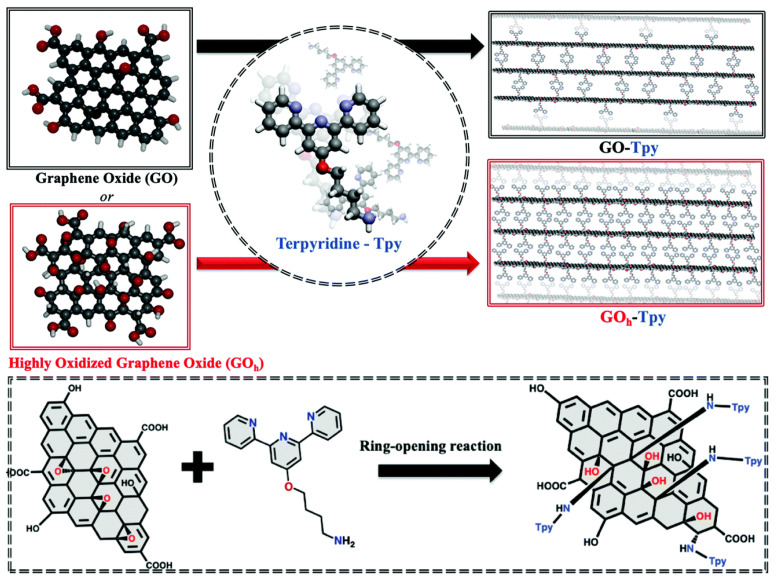
Schematic representation of the functionalization of GO with two levels of oxidation (GO and GO_h_) with Tpy using the ring-opening reaction of epoxides yielding GO–Tpy and GO_h_–Tpy. Reprinted with permission from Ref. [211]. Copyright©The Royal Society of Chemistry 2021.

GO and highly oxidized graphene oxide (GO_h_) with different degrees of oxidation were combined with tridentate terpyridine ligand (Tpy) to form GO, GO_h_, and GO–Tpy as shown in Figure 13. Compared with the prepared GO, GO_h_, and GO–Tpy, GOh–Tpy has the highest adsorption efficiency for heavy metal ions due to the synergistic effect of GO and Tpy components. The maximum adsorption capacity (q_max_) of the GOh–Tpy system for Ni (II), Zn (II), and Co (II) reached 462, 421, and 336 mg g^−1^ at pH = 6, respectively, and showed excellent repair performance, which proved that the GOh–Tpy mixture had an ideal cycle stability, reusability, and easy separation operation [211].

### 3.3. Degradation of Gaseous Pollutants

Exposure to air pollution is one of the five major global human-health risk factors. Photocatalytic oxidation is a promising method for the treatment of environmental pollutants. Nitrogen oxides (NO + NO_2_), benzene, and isopropanol are the three major pollutants that can be commonly found outdoors. Titanium dioxide/graphene hybrid nanomaterials were synthesized by the sol–gel method. Under UV-A irradiation, the presence of isopropanol and benzene will form different free radicals, which improves the removal efficiency under UV-A irradiation. The addition of at least 1.0 wt% of granolol to TiO_2_ can double the photocatalytic efficiency, and the system exhibits more stability under oxidizing conditions compared with pure TiO [212].

**Figure 14:**
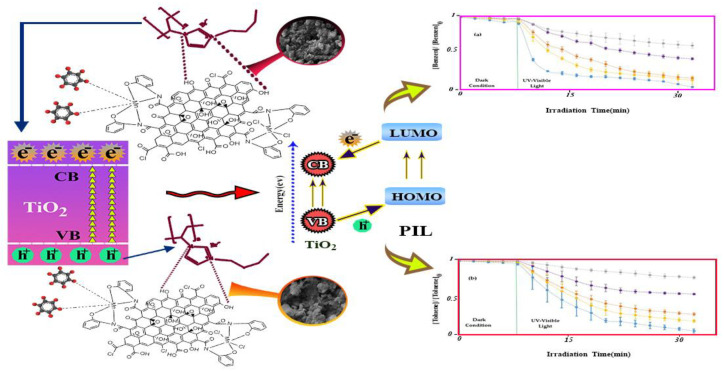
Schematic illustrating photocatalytic degradation with the help of band structures. Reprinted with permission from Ref. [213]. Copyright©2021 by the authors. Licensee MDPI, Basel, Switzerland.

A multi-ionic liquid (PIL)/TiO_2_ composite material directly degrades pollutants through a free-radical mechanism and promotes the absorption and degradation of composite pollutants. Its photocatalytic degradation is shown in Figure 14. PIL/TiO_2_ has high and low concentrations, and its photodegradation rate for benzene and toluene pollutants is also different. When the concentration is high, the photodegradation rate of benzene and toluene pollutants can be obtained at only 59% and 46%, respectively. Meanwhile, the decomposition rates can reach 86% and 74%, respectively, at the low concentration. When the PIL @ TiO_2_/modified graphene oxide (m-GO) is at a high concentration, the photodegradation rate of benzene and toluene can reach a 91% oxidation rate, while PIL @ TiO_2_/m-GO can reach 97% at a low concentration, and the percentage is obtained within 24 min [213].

**Figure 15:**
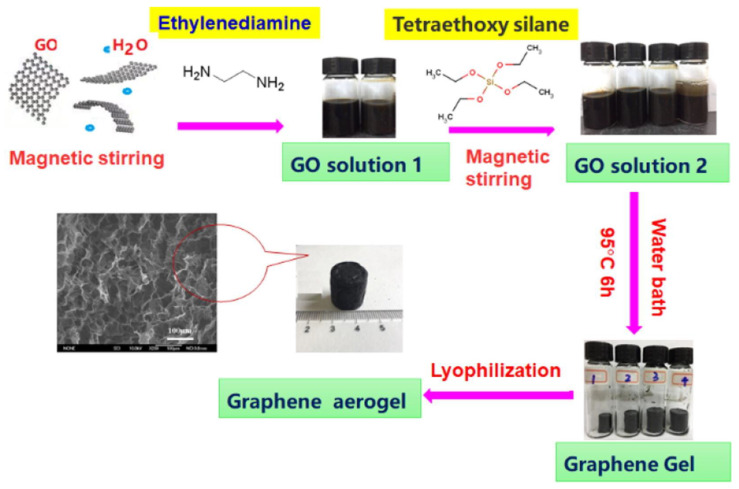
The preparation process of graphene aerogels. Copyright© 2021 The Authors. Reprinted with permission from Ref. [214]. Published by Elsevier Ltd.

A novel high-strength graphene aerogel was prepared by adding tetraethoxysilane (TEOS) to the precursor GO solution, as shown in the Figure 15, which has an ultra-high adsorption capacity for gas pollutants. On this basis, the graphene aerogel modified by SiO2 is more sensitive to benzene vapor, and the adsorption capacity of benzene vapor increases from 201.71 mg/g to 809.1 mg/g. In addition, it can also be used for the separation of benzene–toluene mixtures. This graphene aerogel can be reused after heat treatment at 150 °C. More importantly, the strength and adsorption capacity are not significantly reduced. This shows that the graphite aerogel in the field of indoor pollution gas removal has broad application prospects [214].

## 4. Photocatalytic Reduction of CO_2_

### 4.1. Mechanism

Solar energy will be an important component of future energy improvements for new sustainable development. Research on the photocatalytic reduction of CO_2_ began in 1972 and 1979 when Fujishima and Honda used TiO_2_ as a motor to photocatalyze the reduction of CO_2_ in water [87,215,216]. The reduction and oxidation potential of CO_2_ is matched with the valence band and the conduction-band position of the semiconductor photocatalyst, but most of the semiconductors cannot satisfy this requirement; thus, to achieve CO_2_ reduction and emission reduction in photocatalytic reactions under sunlight, it is necessary to find semiconductors that meet the above conditions.

According to the basic reaction principle, the photocatalytic reduction of CO_2_ has three main steps: (1) the generation of photogenerated electrons by bandgap engineering; namely, the generation of photogenerated charge carriers and the formation of electron-hole pairs; (2) the transfer of photogenerated electrons—this process can be called charge carrier separation and transport; and (3) the reduction of CO_2_ by photogenerated electrons on the surface.

Given that electron-hole pairs combine easily and restrain charge separation to a great extent, effective charge separation is the key to achieve large-scale CO_2_ emission reduction. Graphene is used as electron acceptor/transporter due to its high power function and good conductivity.

In the process of photocatalysis, graphene plays the following roles: (i) reduces photogenerated electrons and hole recombination, (ii) promotes CO_2_ adsorption through π-π conjugation between graphene and CO_2_, (iii) activates CO_2_ molecules, (iv) improves corrosion resistance, (v) enhances surface area and light absorption, which subsequently results in higher photocatalytic activity [217].

### 4.2. Main Optimization Strategies of Graphene

#### 4.2.1. Metal Doping, Non-Metal Doping and Graphene Heterostructure

Since most catalysts are easy to recombine due to the influence of electron-hole pairs in the process of CO_2_ reduction, the catalyst surface activity is reduced, and the production efficiency is low [218]. Graphene doping is an effective method to adjust its electrical properties and expand its applications [219]. Graphene with different properties can be obtained by doping [220,221,222]. Examples include heteroatom doping in graphene, graphene nanosheets (GNS), graphene nanoribbons (GNR), graphene hydrogels, graphene quantum dots (GQD), GO, and rGO [223,224,225,226,227].

Doping is the most feasible and convenient method to adjust the band structure of graphene from semi-metal to p-type or n-type materials [228,229]. Primitive graphene usually shows bipolar characteristics. However, due to different specific needs, we need to make different semiconductors or electronic components, such as those with the above p-type and n-type conductivity, to manufacture logic circuits for industrial applications [230,231].

Graphene doping can be divided into n-type doping, p-type doping and p/n co-doping of single-layer or double-layer graphene. The chemical doping of graphene is realized by attaching heteroatoms to the surface of graphene or by replacing C atoms in graphene. For graphene, it is easier to produce p-type doping through surface adsorption [232]. Exposure of the original graphene in molecules with electron-absorbing groups (H_2_O, O_2_, N_2_, NO_2_, PMMA, etc.) will lead to obvious p-type doping. If the heteroatoms are removed from the p-type doping, the p-type doped graphene will return to the state before the original doping [233].

Chemical doping can effectively open the band gap of graphene [234]. The Fermi point on the doped graphene can be moved up and down depending on the different types of dopants, causing charge separation and easier formation of electron-hole pairs. The external factors that determine the band gap of graphene depend on the surface adsorption energy, lattice displacement doping, and so on [235]. X-ray photoelectron spectroscopy (XPS), angle resolved photoemission spectroscopy (ARPES), potential energy surface scanning (PES), and other methods can be used for the characterization of doped graphene and to detect its properties [236].

As a doped atom, graphene can provide a large number of electrons, which is due to the existence of large π bonds. The electrons in its parallel p-orbitals enter the conduction band, leaving a large number of holes in the valence band, forming an electron-hole pair. The Fermi energy level is close to the bottom of the conduction band. This doping method is p-type doping. In general, graphene has a two-dimensional honeycomb structure, so its surface easily adsorbs some small molecules, such as H_2_O, N_2_, O_2_, and CO_2_. These small molecules will promote graphene to form p-type doping. We can also open the sp^2^ bond of graphene by replacing the position of carbon atoms or other atoms and bonding with carbon atoms to form doping. Because graphene can be prepared in large quantities by chemical vapor deposition (CVD), we can add different reaction sources to it at the end of the production, so that some carbon atoms in graphene can be replaced to form lattice doping. For example, under certain conditions, boron atoms can partially replace carbon atoms, forming p-type graphene. Common p-type doping molecules include fluoropolymer, water, N_2_, NO_2_, O_2_, oxidizing solution, B, Cl, and metal [236]. Khudair and Jappor have proved that the adsorption of CO_2_ on B-doped graphene and double B-doped graphite has strong chemisorption. The strong interaction between single- and double-B-doped graphene composites show that B-doped graphene and double B-doped graphene can catalyze or activate, which indicates that B-doped graphene and double B-doped graphene can be used as effective catalysts for CO_2_ reduction [237].

Individual metals are easy to dissolve in the reaction environment because of their low bulk-binding energies, which have been extensively calculated by Chen et al. In the ORR performance of the 10 metal-doped graphene (M-G) catalysts (light metal Al; semiconductor Si; 3d-metals Mn, Fe, Co, and Ni; 4d-metals Pd and Ag; and 5d-metals Pt and Au) they studied, the metal bound to the graphene had higher binding energy, which shows that the M-G catalyst can be more stable compared with samples without metal dopants [238].

In the study by Min et al., different kinds of metal-doped graphene (for example, N-doped graphene, Ga-doped graphene and co-doped graphene) were found to have different band gaps [239]. The band gap of N-doped graphene was 0.20 eV, that of Ga-doped graphene was 0.35 eV, and that of N-Ga-doped graphene was 0.49 eV. They also had different electron densities. In N-Ga co-doped graphene, N atoms gained more electrons (−1.027 electrons) than N-doped graphene (−0.6 electrons), and Ga atoms lost more electrons (1.75 electrons) than Ga-doped graphene (1.80 electrons). At the same time, it was also found that different doped elements of graphene also have different electronic densities. For example, in the N-Ga co-doped graphene, the N atom gained more electrons (−0.61 electron) than the N-doped graphene (−0.27 electron), and the Ga atom lost more electrons (1.80 electron) than the Ga-doped graphene (1.75 electron).The analysis of different kinds of doped graphene shows that doped graphene has better photocatalytic performance; this also proves that doping has an important effect on the study of the photocatalysis of graphene [240].

**Figure 16:**
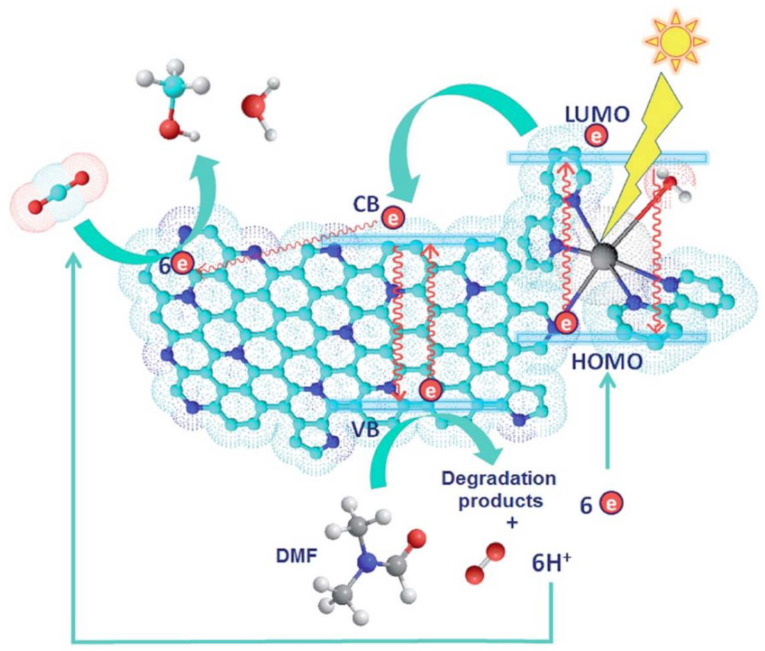
Plausible mechanism of CO_2_ reduction by using a GrN_700_–CuC catalyst. Reprinted with permission from Ref. [241]. Copyright©Royal Society of Chemistry 2023.

As shown in Figure 16, Pawan Kumar et al. report a novel approach for grafting a copper complex onto N-doped graphene. It shows the reasonable mechanism of reducing CO_2_ with a GrN_700_-CuC catalyst. At the same time, in order to verify that the reduction product methanol is produced by CO_2_, they replaced CO_2_ with N_2_ and found that the methanol content is small enough to be ignored. All the results show that the N-doped graphene catalyst has good chemical stability and can be further applied and developed.

As a material with excellent photosensitivity and conductivity, graphene has a good application prospect in the field of photocatalysis and CO_2_ reduction. However, in practical research, it is found that a single-metal material cannot achieve high photocatalytic efficiency, and a graphene heterostructure can effectively solve this problem.

**Figure 17:**
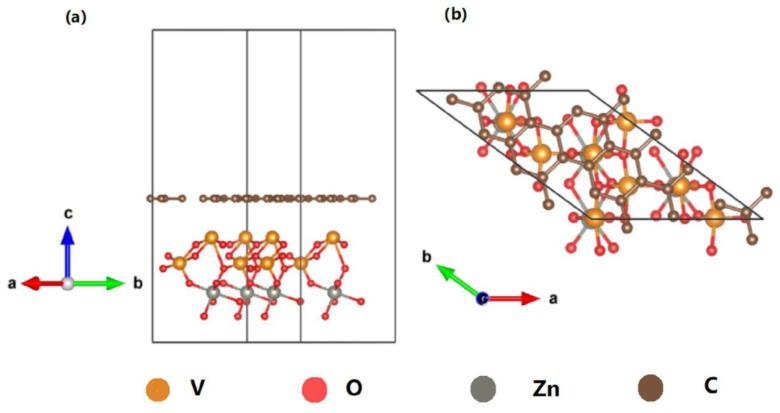
Model for the graphene/ZnV_2_O_6_(001) heterostructure viewed from the side (**a**) and the top (**b**). Reprinted with permission from Ref. [243]. Copyright © 2023 Elsevier B.V. or its licensors or contributors.

First of all, we found that the heterojunction can significantly improve the reaction efficiency in the reduction reaction of CO_2_, because the graphene heterojunction effectively reduces the energy band structure of CO_2_ and can more easily dissociate the C-O bond (750 kJ·mol^−1^) [242]. Doping heterostructures into graphene can effectively improve the function of graphene and significantly reduce its energy band structure. For example, a graphene/ZnV_2_O_6_ heterostructure can reduce its energy band structure to 0.025 eV. This is because heterostructures in graphene can effectively migrate electrons from the interior to the surface. They are excited to graphene through the electrostatic field, promoting the separation of electron-hole pairs and inhibiting the recombination of carriers. In this way, a large number of electrons can be formed on the graphene surface, and a large number of holes can be accumulated on the heterostructure. Such a good combination can greatly promote charge transfer and photocatalytic efficiency [243] (Figure 17).

Zhiling Tang et al. developed the ternary catalyst of rGO-coated Ag/Cu_2_O-octahedron nanocrystals (Ag/Cu_2_O@rGO). In order to obtain materials with more photoelectrons, Tang et al. coated silver nanoparticles on the materials, according to the characteristics of silver nanoparticles with low Fermi energy, and finally obtained more photoelectrons in Cu_2_O. At the same time, because of the high specific surface area and two-dimensional honeycomb structure of GO after sp^2^ hybridization, it can adsorb gas better and enhance activation ability by coating a Ag/Cu_2_O surface. In the end, they concluded from a number of experiments that the ternary heterojunction can effectively reduce the CO_2_ content in photocatalysis and selectively produce CH_4_ [242]. Similarly, Fei Li, Li Zhang, and others also proved that when exposed to visible light (λ4400 nm), the use of a single double-sided Cu_2_O/graphene/TiO_2_ nanotube array (TNA) heterostructure as a separate oxidation and reduction catalyst, which uses anode TNA as the substrate, electrodeposits graphene and Cu_2_O in turn. The results of photoelectrochemical measurements show that the ternary heterogeneous materials display the advantages of each component, improve the photocatalytic performance significantly, and show excellent performance in light absorption and the reduction of electron-hole pair coincidence; in other words, ternary heterogeneous materials are excellent catalysts for photocatalytic reactions [244].

It is concluded from the above-mentioned optimization strategies that the inhibition of electron-hole-to-ground recombination is the key to enhancing photocatalytic efficiency. Graphene-based photocatalytic engineering is an effective way to reduce CO_2_ and convert it into fuel because of its superior photocatalytic properties when combined with metal oxides.

Graphene’s broad absorption spectrum is one of the main reasons for its excellent photosensitivity, and its excellent performance in light absorption is an effective way to improve the efficiency of photocatalysis. Li and his colleagues reported that the addition of graphene to Pt-TiO enhanced the light absorption. In this process, finding the appropriate doping stoichiometry is the key to improving the optical absorption efficiency.

A great deal of research has been conducted on the preparation of highly efficient heterojunction photocatalysts. Rui Sun et al. obtained the heterojunction of a perylene diimide/Graphene-g-C_3_N_4_(PDI/G-CN) nanosheet and demonstrated the potential practicability of an S-type heterojunction in photocatalysis [245].

Radovic et al. have found that the transfer of photogenerated electrons occurs through Valery Karpin sites in graphene after doping and that the surface of the doped graphene has more oxygen vacancies, and realignment occurs. The active sites on the surface provide more sites for CO_2_ reduction [246].

Khaja Mohaideen Kamal, Rekha Narayan, Narendraraj Chandran, and others studied the synergistic enhancement of plasma gold nanoparticles on a TiO_2_-modified N-graphene heterostructure catalyst for CO_2_ reduction for highly selective methane production. Doped graphene itself has excellent photocatalytic performance, and TiO_2_ also plays an excellent role in synergistic catalysis. Properly designed plasma gold nanoparticles electrodeposited onto TiO_2_-modified N-doped graphene (ANGT-x) heterostructure catalysts exhibit significant CO_2_ reduction activity and high methane generation selectivity. Compared with the typical binary Au-TiO_2_ photocatalyst, the electron consumption rate (Repetron) value of the reduction product of ANGT2 is about 742.39 µ mol g^−1^h^−1^,the latter is 60 times more than the former. As far as we know, this is the highest PCO2R rate reported under comparable conditions. These remarkable improvements are attributed to good light selectivity and improved electron transfer dynamics. At the same time, due to the formation of the heterojunction by doping, the components form a seamless interface. Their contact greatly inhibits the recombination of electron-hole pairs and improves the efficiency of charge separation.

#### 4.2.2. Compound Materials

The combination of graphene and semiconductors as a synergistic heterogeneous composite can improve the efficiency of photocatalysis or electrochemical CO_2_ reduction. On the premise that the coupling of CO_2_ reduction and H_2_O oxidation forms a complete photoelectrochemical cycle, graphene can change the conduction-band potential of semiconductors to form graphene-based composites, which leads to the reduction of CO_2_. It has been reported that graphene/WO_3_ nanobelt composites can reduce CO_2_ to CH_4_, in which graphene improves the conduction band of WO_3_ in the composites, while single WO_3_ inherently limits the reduction of CO_2_.

The main preparation methods of graphene matrix composites include physical coating, CVD, electrophoresis, and electrodeposition. The physical coating method is to attach the available graphene or graphene-based composite materials to the target materials without changing their physical and chemical properties. The CVD method has been briefly explained in the above section on chemical doping. Electrophoretic deposition (EPD) is a general processing technology for depositing graphene with controllable thickness and uniform structure on a wide range of substrates [247,248,249].

Herein, we show you the preparation method for TiO_2_-rGO nanocomposites developed by Liu et al. They used GO and TiO_2_ nanoparticles as starting materials to realize the efficient photocatalysis of GO composites through simple and relatively general methods.

It is difficult to reduce CO_2_ efficiently simply by using components as photocatalytic materials. Metal–organic framework (MOF)/GO composites were prepared for CO_2_ capture under flue gas conditions by using the method of Mégane Muschi and Sabine Devautour-Vinot, and its performance greatly exceeds that of pure components. Based on the microporous water-stabilized MIL-91(Ti), a series of CO_2_-capture composites were prepared by in situ and post-synthesis methods. It was observed that 5 wt% GO in situ composites exhibited semi-conductive behavior, whereas the composite was insulating, even though the GO content was high (20 wt%). Therefore, compared with pure MOFs and post-synthesized materials, this composite material absorbs microwave radiation more effectively. Finally, Muschi and Devautour-Vinot reported that CO_2_ desorption under microwave irradiation is faster than direct electrical heating on MOF/GO in situ materials, paving the way for energy-efficient microwave-swing adsorption processes in the future [250].

Graphene itself is a two-dimensional honeycomb material. It is precise because of its structural characteristics of a high specific surface area, which greatly increases its reaction efficiency. Zhuxing Sun and Yun Hang Hu proposed several 3D structure graphene materials that can reduce the accumulation of graphene sheets and help improve the photocatalytic efficiency of graphene-based materials. They found that the exothermic reaction between an alkali metal and inorganic carbon compounds provides an ideal solution for the efficient utilization of CO_2_ and the cost-effective production of 3D graphene. In many reactions, in order to solve the problem of building up micro-scale 3D graphene materials, it is necessary to form alkali metals and CO_2_ graphene at the same time and use them as original templates. Etching graphene by CO_2_ is the key project to control the formation of porous surface structures, so it also provides a good scheme for constructing various excellent 3D graphene materials [251].

The current research on graphene-based composites is aimed at reducing CO_2_ emissions. There are many types of materials in this field, but they are mainly based on graphene for the mixing of three or other components. Barbara Szczęśniak et al. have studied three-component GO/ordered mesoporous carbon/metal organic matrix composites to address the need for expensive organic binders in the field of composites, and they found that the three-component composite also contributes to the formation of MOF crystals during air raids on mesoporous networks and CO_2_ recovery [252].

#### 4.2.3. Bandgap Engineering

Graphene has a strong s bond between its two carbon atoms due to its “sp^2^” hybridization, which greatly enhances its structural strength and flexibility. Based on this hybrid, graphene “sp^2^” hybrid-connected carbon atoms can be tightly stacked into a single-layer two-dimensional honeycomb lattice structure. Bandgap engineering can effectively adjust the bandgap of graphene, and it plays a role as a charge-transfer carrier to enhance the efficiency of electron transfer and to increase the reduction rate of CO_2_ [144].

The translational symmetry of the original graphene is broken, that is, when two equivalent atoms in the original cell become unequal, a band gap will be generated. The experiment of introducing a band gap into the original graphene includes growing graphene on some substrates, applying an external bias voltage, generating graphene in the form of narrowband graphene, and perforating graphene to form a “graphite nanonet” [253,254].

Through a lot of research on graphene bandgap engineering [255,256,257,258,259,260,261,262], it is found that there are two main methods to change the band gap of graphene: (1) By triggering the quantum confinement effect to open the obvious band gap, which requires greatly reducing the size of graphene to 10 nm through nano-level operations to form nanoribbons. (2) By the adsorption or coating of some substances on the surface of graphene to affect its reaction, thereby changing the electronic structure of graphene. In practice, the first method is not often used in practical applications because it is affected by the nanoribbons and bandgap openings [263,264].

It has been proved by experiments that if nanopores or molecularly modified graphene are introduced into the original graphene, the required translational symmetry can be removed, thus opening the band gap. Doping some elements into graphene can open the band gap. For example, the hydrogenation of graphene can effectively adjust the band gap of graphene. In addition, some graphite intercalation compounds (GIC) are outstanding in inducing superconductivity [265].

At the same time, because the volatile organic compounds adsorbed on the surface of the catalyst combine with the lattice oxygen on the surface of the catalyst, the catalyst surface generates oxygen holes and is reduced, and the catalyst is oxidized by filling the oxygen vacancies with the dissociated adsorbed oxygen. The resulting graphene derivatives, such as GO and rGO, have the characteristics of insulators and similar semiconductors in terms of photocatalysts, resulting in wide band gaps. GO has a limited band gap due to the two-dimensional grid generated by its “sp^2^” and “sp^3^” hybridization. Therefore, the proportion of operating hybrid-bonding atoms can effectively adjust the broad band gap of GO, transforming it from an insulator to a semiconductor and into a metal similar to graphene. The oxidation functional groups around GO can operate through H_3_PO_4_. The amount of H_3_PO_4_ is the key to the operation affecting the oxidation functional groups, which leads to a tuned band gap that is well aligned with the CO_2_/CH_3_OH redox potential (vs. NHE, pH = 7.0) and shows an improved activity of CO_2_ photoreduction to methanol. The conversion of CO_2_ to methanol requires stretching the band gap of GO so that there is a CH_4_ production orientation in the CO_2_ conversion. After much research, it has been found that there are many oxygen groups on the surface of GO, which can effectively stretch the band gap of GO. This stretching regulates the oxidation and reduction potential of CO_2_ to match the valence band and the conduction band and promotes the generation and excitation of photogenerated electrons, which can migrate to the surface more effectively and induce CO_2_ reduction.

Sokal reported on the bandgap engineering of graphene. We know that photocatalysis produces photogenerated electrons, while doped graphene exhibits better charge separation properties. In his paper, Sokal pointed out that the graphene doped with TiO_2_ can separate the electron and hole more effectively and keep them in different positions. Tang and colleagues have also demonstrated that graphene-coated metals exhibit rapid CO_2_ reduction over a longer period of time [266].

**Figure 18:**
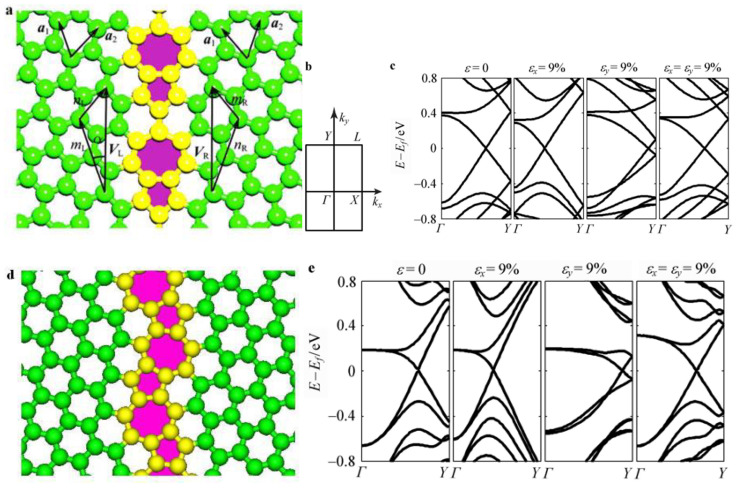
GB structure and band structure of symmetrical polycrystalline graphene. (**a**) Detailed GB structure of armchair-tilt (θ = 21.8°) graphene. (**b**) The first Brillouin zone of all structures and special K points used to calculate the band structures. (**c**) The corresponding band structures for the structure in (**a**) with different types of strains. (**d**) Detailed GB structure of zigzag-tilted boundary with θ = 27.8°. (**e**) The corresponding band structures for the structure in (**d**) with different types of strain. Reprinted with permission from Ref. [267]. Copyright©2023 Springer Nature Switzerland AG.

In order to effectively adjust the band gap structure of graphene, Wu et al. calculated the energy band structure of polycrystalline graphene through the density functional theory (DFT). The results showed that even under the condition of external stress, the grain boundaries (GBs) with symmetrical polycrystalline graphene still have zero band gap, while some asymmetric GBs can also open the band gap, which can be adjusted by external stress (Figure 18). The discovery of this study plays an important role in the further study of graphene bandgap engineering.

## 5. Summary and Outlook

Graphene and its derived materials are functional materials that have emerged in recent years. They have also been involved in a series of breakthrough discoveries in the field of photocatalysis and pollutant degradation and have spawned the creation of many new photocatalytic materials and applications. So far, many of their advantages have been exploited and utilized by people, and there will be more excellent potential applications in the future. However, there is no denying that graphene also has some shortcomings to be overcome, mainly involving the following aspects:

(1) Graphene semiconductor materials have been deeply studied in the field of photocatalysis, and they have shown good photocatalytic material properties when combined with many precious metals. They can reduce the bandgap energy and improve the electron-hole yield. They are a good choice to reduce the greenhouse gas CO_2_ and to obtain chemical fuel–metal composite graphene materials similar to CH_4_ that can be used. However, as a new material, at the environmental level, the economic cost and environmental resource cost of using precious metals to improve the photocatalytic effect are still huge. If some metals are not properly treated, this may cause pollution to the environment and indirectly increase the cost of the subsequent treatment of materials. Metal–graphene composites with low cost and high photocatalytic energy efficiency remain to be found. TiO_2_ can efficiently utilize ultraviolet light. Although many TiO_2_ composite graphene materials can expand the range of the photocatalytic reaction light wave, the effect still has a large room for improvement. Making full use of the visible-light band will greatly improve the photocatalytic energy efficiency. In graphene bandgap engineering, the band gap can be opened by reducing the size of the graphene material [268]. This method does provide a certain bandgap engineering idea, but in view of the edge structure of the bandgap opening and its strong chemical modification sensitivity, the application of this method is still challenging. 

(2) By constructing two-dimensional and three-dimensional graphene composites, or by constructing porous materials, the high surface-area ratio, optical properties, and mechanical structure stability of graphene can be maximized. These characteristics can increase the photocatalytic reaction area, CO_2_ absorption rate, water decomposition efficiency, etc. and significantly improve the photocatalytic effect. Furthermore, there will be no environmental disadvantages of noble-metal composite graphene photocatalytic materials. However, to be economically viable, researchers need to continue to push for lower material costs so that they can be produced on a large scale and use solar energy efficiently. At the same time, there are also some problems to be addressed in the construction of heterojunction structures by organic non-metallic graphene materials. For example, the existence time of two carriers excited by quantum light is short and the photocatalytic efficiency is low, and it is difficult to avoid agglomeration when graphene nanomaterials are introduced. The reaction site of photocatalysis is not positively correlated with the amount of material input. According to the semiconductor properties of graphene, the development and production of highly active graphene-based photocatalysts can be further studied. For different types of graphene-based materials (open-bandgap graphene materials, zero-bandgap perfect-single-layer graphene, etc.), their electrical properties may have unexpected effects in their applications. At the same time, the application of graphene-based composites in photochromic or electrochromic fields is not mature. Metal oxide-based composite graphene materials or glass-fiber composite graphene may make full use of the semiconductor properties of graphene to participate in the redox process of electrochromic materials and to promote the development of products such as “smart glass”. Furthermore, in catalytic reactions, due to the easy recombination effect of electron-hole pairs, we need to introduce heteroatoms to adjust the band structure of graphene and to reduce the recombination rate of electron-hole pairs. For different types of doped graphene, we have made a detailed overview of metal-doped graphene and non-metal-doped graphene and have given certain conclusions in terms of their basic electronic structure, catalytic active site, and morphology. However, the structural characteristics and functions of doped graphene still need further discussion and research.

(3) The use of graphene for pollutant degradation has given rise to more graphene-based composites for environmentally friendly cleaning purposes or to enhance the performance of graphene. However, most forms are just composite materials, composite membranes, composite hydrogels, etc., and few become a green and clean treatment system. They have shown good performance in the laboratory, and some can even achieve a 100% removal rate. They also show good recyclability after an adsorption cycle and will not cause secondary pollution to the environment. However, most of them only have good adsorption for individual pollutants, and the removal efficiency for most of the pollutants not mentioned in the discussion are still unknown. In addition, it is also necessary to consider whether the laboratory simulation environment or sampling can fully match the real environment. The environment of different regions is also under different conditions. How to carry out the next industrialization or commercialization step is still a question mark. The cost of material manufacturing or cleaning system construction is one of the issues we need to consider. Nonetheless, if there is a better application than the current choice, that would be a small success.

(4) Some defects of graphene-based photocatalyst lead to its low recycling rate, which is rarely considered in most of the current literature. For example, the synthesis of N-doped graphene-based photocatalysts will produce vacancy defects at the same time, which significantly affects the activity of photocatalytic materials. For environmental protection and sustainable development, how to maintain the stability and service life of graphene-based photocatalysts is a direction worthy of study.

(5) Graphene has become an important photocatalytic material in the 21st century. In this article, we discuss in detail the bandgap engineering for constructing graphene, which can fully utilize its role as a charge carrier and improve electron transfer efficiency and CO_2_ reduction rate. These methods include adding graphene to the substrate, applying an external voltage, producing graphene in the form of narrowband graphene, and using “graphite nanogrids”. The specific graphene band gap provides enormous room for progress in photocatalytic reactions and will be further expanded and developed in the future field of photocatalysis. Single-layer pure graphene sheets lack hydrophilic functional groups, and the preparation of semiconductor/pure graphene composites is extremely challenging. At the same time, as a 2D material, it is still worth exploring how to achieve large-area interface dynamics with other non-2D materials or to load various components to achieve charge separation in space.

(6) Studies suggest that cytotoxicity mainly depends on the physical and chemical properties of nanomaterials. Surface-doped chemical or biological molecules will also affect the expression of properties to some extent. Nowadays, many graphene products exist in the market with smart devices and sensors, which also creates a problem. Graphene-based materials have toxic effects. When put into use, graphene nanoparticles have a potential risk of exposure to air, which will cause certain irritation to the human body and affect health. In order to give full play to the commercial value of graphene nanocomposites in practical market applications, more in-depth research still needs to be conducted in this area.

## Data Availability

Not applicable.
